# Symposia Abstracts

**DOI:** 10.1080/24740527.2021.1914213

**Published:** 2021-04-22

**Symposium Chair**: Kathryn Birnie, PhD RPsych, Assistant Professor, Department of Anesthesiology, Perioperative and Pain Medicine, University of Calgary, Calgary, AB, Canada; kathryn.birnie@ucalgary.ca; @katebirnie

**Symposium Abstract**:

The amount of scientific output globally is increasing at an exponential rate. A resulting problem from this science overload is that knowledge production has become increasingly granular, repetitive, and insular. This insularity in scientific inquiry isolates pain research from the realities of clinical practice, lived experience, and the broader social context; thus, creating a substantive disconnect between knowledge generation and its implementation. Addressing this concern is no more critical than in the time of the COVID-19 pandemic with an abundance of rapidly produced science and misinformation. The poor uptake of scientific knowledge of pain into clinical practice and policy should be considered a full systems failure. Scientists are not supported, equipped, or rewarded for translating their findings, leading to substantive waste in the production and reporting of research. A critical gap in pain research has been the omission of people with lived experience with pain, such as pediatric and adult patients, and family members. Patient engagement, also referred to as public involvement in health research has value for improving research relevance, design, efficiency, and implementation while also resulting in reduced research waste. Integrated knowledge translation engages stakeholders and potential research knowledge users throughout the entire research process. This symposium will show how people with lived experience are being engaged as stakeholders in integrated knowledge translation from bench science to clinical practice and policymaking across the knowledge-to-action cycle (new knowledge creation, synthesis, and implementation). (*This session was previously accepted for the cancelled CPS 2020 meeting in Calgary).

**Speaker 1**: Nader Ghasemlou, PhD, Queen’s University, Department of Anesthesiology and Department of Biomedical & Molecular Sciences, Kingston, ON, Canada, nader.ghasemlou@queensu.ca, @ghasemloulab

Bradley J. Kerr, PhD, University of Alberta, Department of Anesthesiology and Pain Medicine, Edmonton, AB, Canada, bjkerr@ualberta.ca

**Speaker 1 Abstract Title**: Of mice and men: Strategies to bridge the gap between the laboratory bench and patient populations

**Speaker 1 Abstract**: Our two laboratories have developed and established bonds with patient advocacy groups and patients themselves to better integrate their voices into our research programs. This includes work with the CIHR-SPOR Chronic Pain Network, the Multiple Sclerosis Society of Canada, and the Multiple Sclerosis Society of Canada Alberta/NWT and BC Divisions. Through various initiatives, members of our teams engage with patient-partners to identify new avenues of research and with the public, through lab tours and speaking engagements, to provide insight into the strategies used in the laboratory to study disease related pain. In this presentation, we will outline our experiences with these different outreach initiatives and discuss some of the advantages and benefits we have observed through these opportunities. We will focus on some of the major outcomes these outreach opportunities have had for trainees in our respective research programs.

**Speaker 2**: Kathryn Birnie, PhD RPsych Assistant Professor, Department of Anesthesiology, Perioperative and Pain Medicine, University of Calgary, Calgary, AB, Canada; Assistant Scientific Director, Solutions for Kids in Pain (SKIP); kathryn.birnie@ucalgary.ca; @katebirnie

**Speaker 2 Abstract Title**: Mapping scientific evidence to patient-identified priorities in pediatric chronic pain to inform policy and practice

**Speaker 2 Abstract**: In late 2018, our Partnering For Pain team completed a James Lind Alliance Priority Setting Partnership that engaged hundreds of Canadians with lived experience with pediatric chronic pain, family members, and healthcare providers to identify the Top 10 priorities for pediatric chronic pain research and care. The final Top 10 list prioritizes chronic pain prevention, impact, and treatment, as well as delivery, access, and coordination of care. A key knowledge translation goal is to ensure that these patient-oriented priorities are integrated and addressed within relevant pain policies, strategic priorities, and clinical practice. This talk will present an Evidence and Gap Map, created in partnership with patient partners, to facilitate evidence-informed decision-making in pediatric chronic pain. Evidence and gap maps utilize rigorous systematic review methodology to create a schematic representation of the types of interventions and relevant outcomes to make existing evidence accessible to researchers, decision-makers, and research funders. From 4168 abstracts identified in database searches, 50 systematic reviews (including 2 clinical practice guidelines) of any treatment modality in pediatric chronic pain were reviewed and rated for quality using the AMSTAR-2 appraisal tool. The resulting evidence and gap map identified critically low to high quality reviews crossing pharmacological, psychological, and physical treatments. Evidence gaps exist for interventions that address patient-identified priorities related to prevention, school/education, physical treatments, mental health, and acute pain flares.

**Speaker 3**: Linda Wilhelm, President, The Canadian Arthritis Patient Alliance; Midland, Kings County, NB, Canada; lindaa.wilhelm@gmail.com

**Speaker 3 Abstract Title**: Changing Directions, Patients Influencing Policy

**Speaker 3 Abstract**: The voice of chronic pain patients were not being heard in the context of the opioid crisis. Policies were being developed that had unintended consequences for people living with pain. Together with other patient organizations, the Canadian Arthritis Patient Alliance (CAPA) wrote and met with government. CAPA surveyed our network and brought these perspectives to the Minister of Health. The pain community attended the 2018 Opioid Summit, patients provided their input into new policies and ultimately the Government of Canada’s Canadian Pain Task Force was announced with 3 of 8 members being people with lived experience. This talk will provide first-hand perspective on the engagement of patients within these pain-related policy initiatives.

**Learning Objective 1**: To demystify how basic scientists can effectively engage with patients and patient organizations to improve pain research design, knowledge translation, and training.

**Learning Objective 2**: To learn evidence and gap maps as a useful and rigorous methodology to facilitate integration of patient-identified research priorities with existing scientific evidence to guide researchers, policymakers, decision-makers, and health research funders.

**Learning Objective 3**: To understand how patients are playing a central and effective role in putting chronic pain on the national health agenda, in part, to address the unintended consequences of opioid crisis policy.
