# Supplementary material for: Symposia Abstracts
Source: Can J Pain. 2021 Apr 22;5(2):A3–A33. doi: 10.1080/24740527.2021.1914213 (PMC8210871; doi:10.1080/24740527.2021.1914213)
Supplement: Related texts

# Abstract: When Public Health Crises Collide: Managing Cancer Pain Amidst the North American Opioid Crisis and COVID-19 Pandemic

**Authors:** Sitara de Gagne, Judith A. Paice, Perri R. Tutelman, Lynn R. Gauthier

**Affiliations:** a Patient Partner, Toronto, ON, Canada; b Feinberg School of Medicine, Cancer Pain Program, Northwestern University, Chicago, IL, USA; c Psychology and Neuroscience, Dalhousie University, Halifax, NS, Canada; d Department of Family and Emergency Medicine, Universitè Laval, Québec, QC, Canada

## Abstract

**Symposium Chair**: Sitara de Gagne, Patient Partner, Toronto, Ontario, Canada, sitaradegagne@hotmail.com

**Symposium Abstract**: North America is in the midst of a public health emergency related to opioid use, referred to as the “opioid crisis”. Opioids play an important role in the management of cancer pain. However, the crisis and subsequent mitigation efforts (e.g., dose restrictions, forced tapers) have had unintended consequences, such as reluctance of patients to take and clinicians to prescribe opioids, and stigma felt by patients and expressed by providers. With the current global pandemic, cancer patients are faced with a crisis within a crisis, with increased opioid-related harms, re-routed cancer services resulting in fragmented or diminished access to care, and reduced opioid prescribing. This workshop will describe the nature and extent of the problem of inadequate cancer pain management amidst the dual public health emergencies of the North American opioid crisis and COVID-19 pandemic across contexts (e.g., United States, Canada), age groups (e.g., children to older adults) and perspectives (e.g., providers, researchers, patients and caregivers). First, J. Paice will discuss the challenge of cancer pain management within the United States opioid epidemic and COVID-19 and evidence-based strategies to mitigate opioid-related risks. Then, L. Gauthier will describe the Canadian response, including the outcomes of 2 national stakeholder meetings and findings from a Delphi study to identify key research questions to drive this field forward. Next, P. Tutelman will present quantitative and qualitative data outlining barriers to pediatric cancer pain management amidst the crises. Finally, S. de Gagne will provide a patient perspective as the parent of a childhood cancer survivor.

**Speaker 1**: Judith A. Paice, PhD, RN, Northwestern University, Feinberg School of Medicine, Cancer Pain Program, Chicago, IL, USA, J-Paice@northwestern.edu, @JPaicePhD

**Speaker 1 Abstract Title**: Balancing Cancer Pain Management During the Time of an Opioid Epidemic

**Speaker 1 Abstract**: Cancer pain is a complex, bio-psycho-social-spiritual phenomenon with causes ranging from the direct effects of the tumor, to the consequences of cancer treatment, along with pre-existing painful conditions. Throughout the trajectory of cancer care, opioid use is often indicated and in fact, it may be unethical to limit or prohibit the use of opioids when pain is severe. While many patients can safely use these agents, the intersection of the unprecedented opioid epidemic, COVID-19 pandemic, and cancer pain further complicates care. Although prescription opioids were originally the source of diversion and misuse during the early years of this epidemic, currently monthly initial prescriptions are decreasing. This is in part due to enhanced education of prescribers but an unintended consequence of attention to this epidemic is that a large number of prescribers are no longer prescribing opioids, potentially limiting access to opioids for those in pain, including people with cancer. Oncologists face the significant challenge of providing cancer pain control that is safe and effective, while limiting individual risk for abuse or overdose and keeping the community free of diverted substances. Most oncology providers report inadequate training in chronic pain principles and in managing addiction. The COVID-19 pandemic has further exacerbated the challenge of cancer pain management by reducing access to integrative and interventional therapies and yielding pain drug shortages. Risk assessment and mitigation measures can be incorporated within oncology care to enhance effective pain management while reducing the potential for harm.

**Speaker 2**: Perri Tutelman, BHSc. (Hons), Dalhousie University, Psychology and Neuroscience, Halifax, NS, Canada, ptutelman@dal.ca, @PerriTutelman

**Speaker 2 Abstract Title**: Children’s Cancer Pain Management in the Era of the Opioid Crisis: Attitudes, Beliefs, and Barriers to Effective Treatment

**Speaker 2 Abstract**: Children and their parents consistently report that pain is one of the most common and distressing symptoms of a cancer diagnosis. While there are effective pharmacological, psychological, and physical strategies to alleviate cancer-related pain, many children continue to report undertreated pain across the cancer continuum. This presentation will provide an overview of the problem of poorly managed pain in children with cancer and the unique pain management barriers this population faces in the era of an opioid crisis and global pandemic. First, data outlining the prevalence and characteristics of pain in children with cancer across the disease trajectory, and the state of the evidence for managing pain in this population, will be presented. Next, the role of family-level factors in the management of children’s cancer-related pain will be reviewed, including results on parents’ concerns regarding the use of pain medication for the treatment of their child’s cancer pain (over 60% agreed that pain medication was addictive and approximately 20% believed that children who take pain medication may learn to take drugs to solve other problems). Unique barriers to cancer pain management in the survivorship phase will be highlighted using data from a qualitative study of childhood cancer survivors (ages 8-18 years) and their parents. Areas for future work, including patient and family education and the need for high quality pain trials in this area will be discussed.

**Speaker 3**: Lynn Gauthier, PhD, Université Laval, Department of Family and Emergency Medicine, Québec, QC, Canada, lynn.gauthier@crchudequebec.ulaval.ca, @docpeper

**Speaker 3 Abstract Title**: Managing cancer pain amidst the opioid crisis and the COVID-19 pandemic: The Canadian response to advancing the state of the evidence and safeguarding access to care

**Speaker 3 Abstract**: The opioid crisis is fuelling inadequate cancer pain management. Prior to the pandemic, media reports, editorials, and emerging US-data described reduced prescribing, opiophobia, stigma, and self-harm, prompting professional societies to warn policy makers against denying pain control. Unfortunately, empirical data documenting the extent and impact of the crisis in Canada are unavailable. This presentation will describe a CIHR-funded, cross-Canada knowledge dissemination and research planning initiative. Its aims were to elucidate the implications of the opioid crisis on cancer pain management in Canada and develop a future research agenda to investigate the impact of the crisis and identify ways to optimize pain management across the disease continuum and the lifespan amidst the crisis. A multidisciplinary team of 41 people with lived-experience, patient advocates, clinicians, and researchers with expertise in pain, oncology, palliative and supportive care, and addictions came together at two 2-day meetings in Vancouver and Montreal, in September, 2019. Topics included the clinical realities of managing cancer pain amidst the crisis, patient and caregiver lived experience, at-risk populations (children, older people, women), genetics, psychological comorbidities, substance misuse, cognitive impairment, other treatments (e.g. non-pharmacological treatments, cannabinoids), and systems and economic factors. Discussions highlighted major knowledge gaps, divergent perspectives on approaches to care, and substantial regional differences, including inconsistencies in reporting of harm outcomes, and systems and regulatory impacts. Results of a Delphi study identifying key research questions and recommendations to ensure access to adequate pain management during the pandemic for people with cancer at high risk of infection will be discussed.

**Learning Objective 1**: To describe the problem of inadequate cancer pain management amidst the North American opioid crisis.

**Learning Objective 2**: To understand the impact of the North American opioid crisis and COVID-19 pandemic on cancer pain management across the lifespan from various perspectives (e.g., providers, researchers, patients and caregivers).

**Learning Objective 3**: To identify evidence-based strategies and national initiatives aimed at improving cancer pain management during the current North American opioid crisis.

# Abstract: Management of chronic pain in the era of COVID-19: Implications for pain clinics, patients’ reality, and potential solutions

**Authors:** Manon Choinière, Mary Lynch, Anaïs Lacasse, Patricia Poulin

**Affiliations:** a Centre de recherche du Centre hospitalier de l’Université de Montréal (CRCHUM), Montréal, Québec, Canada; b Département d’anesthésiologie et de médecine de la douleur, Faculté de médecine, Université de Montréal, Montréal, Québec, Canada; c Department of Anesthesia, Pain Management & Perioperative Medicine, Psychiatry and Pharmacology, Faculty of Medicine, Dalhousie University, Halifax, Nova Scotia, Canada; d Département des sciences de la santé, Université du Québec en Abitibi-Témiscamingue, Rouyn-Noranda, Québec, Canada; e Ottawa Hospital Research Institute, Ottawa, Ontario, Canada

## Abstract

**Symposium Chair**: Manon Choinière Ph.D., Centre de recherche du Centre hospitalier de l’Université de Montréal (CRCHUM), Montréal; Département d’anesthésiologie et de médecine de la douleur, Faculté de médecine, Université de Montréal, Montréal, Québec, Canada, manon.choiniere@umontreal.ca

**Symposium Abstract**: The COVID-19 pandemic affects disproportionately high-risk and vulnerable individuals such as those living with chronic diseases, older adults, in addition to socially and economically deprived populations. Public health restrictions and pandemic-related stress thus added to the pre-existing physical, psychosocial and financial burden that is known to be associated with chronic pain (CP). In fact, since the beginning of the pandemic, confinement measures have limited access to multidisciplinary pain clinics and many other types of management options (e.g., physical therapy, massage, psychological counselling, self-help groups). Challenges surrounding access to various drugs (e.g., opioids, antimalarial drugs used in arthritis or corticosteroids), and persons living with CP who are prescribed opioids were even identified as a vulnerable group in the COVID-19 pandemic because the context makes it difficult to adhere to all prescribing and follow-up opioid guidelines. Finally, fear of going to healthcare appointments and self-medication were observed. This symposium will serve as a discussion platform about the impacts of the COVID-19 pandemic on CP management in Canada. Based on the results of empirical studies conducted during both waves of the COVID-19 pandemic, concrete solutions to improve access and continuity of care for Canadians living with CP during the pandemic and beyond will be put forward.

**Speaker 1**: Mary Lynch Md FRCPC, Department of Anesthesia, Pain Management & Perioperative Medicine, Psychiatry and Pharmacology, Faculty of Medicine, Dalhousie University, Halifax, Nova Scotia, Canada, mary.lynch@dal.ca

**Speaker 1 Abstract Title**: Implications for pain clinics: Results of a National Survey

**Speaker 1 Abstract**: As the result of public health authority responses to the COVID-19 pandemic, pain clinics had to cease providing in-person appointments to reduce contact between patients and staff. We were concerned that ceasing in-person pain services exacerbated the daily hardships already faced by Canadians living with CP. A survey of Canadian adult multidisciplinary pain clinics was conducted to determine impacts on medical and allied healthcare services, and the strategies used to deliver care to patients during the COVID-19 pandemic. Responses received from 17 adult pain clinics across Canada found multidisciplinary pain clinics had to cease or significantly reduce in-person patient contacts during the COVID-19 pandemic and responded by offering telehealth options. In spite of their efforts, patients were found to be waiting longer and had lost access to usual care. Increased levels of pain, stress, and medication use, particularly opioids and cannabinoids, were reported. It was concluded that access to adaptable and innovative technologies, such as telehealth are essential in the care of the one in five Canadians living with CP during times of crises, and must be included as a vital component of a comprehensive Canadian Pain Strategy.

**Speaker 2**: Anaïs Lacasse Ph.D., Université du Québec en Abitibi-Témiscamingue, Département des sciences de la santé, Rouyn-Noranda, Québec, Canada, anais.lacasse@uqat.ca

**Speaker 2 Abstract Title**: Impact of the COVID-19 pandemic on the pharmacological, physical and psychological treatments of pain

**Speaker 2 Abstract**: Careful balance between pharmacological, physical and psychological treatments is recognized as the optimal approach for CP management. For many people living with CP, that equilibrium was disrupted by the COVID-19 pandemic and resulted in pain symptoms deterioration. Lack of access to clinics and exercise facilities because of the pandemic-related public health safety measures was a major problem and people living with CP needed to compensate for having to stop their usual treatments. Increase use of opioids or cannabis were also reported. In this presentation, current quantitative and qualitative data about the impact of the COVID-19 pandemic on the pharmacological, physical and psychological treatments of CP will be discussed. Attendees will also have an opportunity to hear about factors associated with disruption in pain treatment during the pandemic such as sex, age, pain care providers, or province of residence. The significance of data from empirical studies conducted during both waves of the COVID-19 pandemic will be harnessed to propose concrete interventions to better support individuals living with CP.

**Speaker 3**: Patricia Poulin Ph.D. C.Psych., Ottawa Hospital Research Institute, Ottawa, Ontario, Canada, ppoulin@toh.ca

**Speaker 3 Abstract Title**: Improving access to chronic pain through an online pain portal supporting a flexible progressive stepped care approach anchored in recovery principles

**Speaker 3 Abstract**: Stepped Care 2.0 is a flexible progressive stepped care approach anchored in recovery principles and designed to improve access to mental health and substance use care. Using a brief assessment and continuous outcome monitoring, Stepped Care 2.0 matches people seeking care to different interventions that vary in intensity depending on their goals, preference, and readiness for change. This model is endorsed by the Mental Health Commission of Canada and is the underlying framework of Wellness Together Canada, a Health Canada-funded online portal providing access to mental health and substance use support for all Canadians. Stepped Care 2.0 has been adapted for CP management at The Ottawa Hospital Pain Clinic, leading to an elimination of wait-time to access interprofessional care following an initial physician consultation. Multiple stakeholders are now collaborating to develop a national Pain Portal that will provide virtual care education and treatments for youths and adults living with CP, along with providing the necessary infrastructure to evaluate various virtual care interventions. Connection with the Wellness Together Canada portal will allow for leveraging of 24/24 7/7 mental health and substance use support, ensuring more efficient use of our health care resources. Next steps in the creation of this portal will be discussed, along with opportunity for engagement.

**Learning Objective 1**: Learn how Canadian multidisciplinary pain clinics responded and adapted to the COVID-19 pandemic.

**Learning Objective 2**: Understand how the COVID-19 pandemic affected the pharmacological, physical and psychological treatment of CP.

**Learning Objective 3**: Discuss concrete solutions to improve access to pain relief and maintain continuity of care during the pandemic and beyond.

# Abstract: Cannabis and pain: putting the horse back before the cart

**Authors:** Tania Di Renna, Martha Glenny, Karen Ng, Hance Clarke

## Abstract

**Symposium Chair**: Tania Di Renna, MD, FRCPC, Department of Anesthesia, University of Toronto; Toronto Academic Pain Medicine Institute, Women’s College Hospital, Toronto, Ontario, Canada, tania.direnna@wchospital.ca

**Symposium Abstract** (250 words or less): Despite limited clinical evidence, the use of cannabis for management of pain has increased significantly. A recent Canadian national survey indicated that 13% respondents aged 16 and older indicated that they had used cannabis for medical purposes (1). In a cross-sectional survey of Canadian medical cannabis users, (44.8%) endorsed using it for ‘pain relief’ and (15.1%) for ‘mental health’ (2). This is one of the first times in Canadian history where a medication has been widely used for a variety of conditions without actual guiding evidence. With the legalization of recreational cannabis in 2018, Canadians are now able to easily access cannabis via multiple channels, bypassing clinician guidance. The world is looking to Canada to lead cannabis related research and to develop clinical programs that will better inform the care we provide for our patients with pain.

This symposium will address:

1. Patient perspective in navigating the paradigm of medical cannabis and discuss the barriers faced to accessing clinician guidance
2. Programmatic initiatives that addresses knowledge gaps and highlights harm reduction strategies to empower patients with an understanding of the risks and potential benefits of cannabis for pain.
3. The future of Cannabis Research: Present current academic pain research initiatives and discuss what Canadians are consuming, evolving evidence and areas of need.

**Speaker 1**: Martha Glenny, Patient Experience, Advisor, Toronto, Ontario, Canada

mlglenny@icloud.com

**Speaker 1 Abstract Title**: Navigating Medical Cannabis: The Patient Perspective

**Speaker 1 Abstract**: Martha will discuss the patient journey and provide her perspective on navigating cannabis use in the current system. Her perspective will include the barriers to finding trustworthy information and unbiased clinicians who have helped guide her. She will describe her experience(s) interacting with an academic pain practice. To avoid a completely didactic session, Martha will engage in a mock virtual interview with both the pharmacist and physician on the symposium panel.

**Speaker 2**: Karen Ng BScPhm, PharmD, ACPR, Toronto Academic Pain Medicine Institute (TAPMI), Women’s College Hospital, Toronto, Ontario, Canada, karen.ng@wchospital.ca

**Speaker 2 Abstract Title**: Enhancing Knowledge, Mitigating Patients Harms: Standardizing Safer Cannabis Practices and Patient Education

**Speaker 2 Abstract**: Since the Cannabis Act came into act on October 2018, Canadians have access to cannabis through multiple channels. For many, the line separating recreational and medical use blurs and this creates questions around the safety and efficacy of cannabis. In this rapidly evolving world of cannabis, clinicians have to consider how to help patients navigate the healthcare system challenges. s. As a large multi-site academic pain institute, our patients are interested in learning about our position on cannabis’ role in pain, its evidence and risks. This presentation will describe two programmatic initiatives 1) development of a interdisciplinary position paper that strives to reduce clinical practice variability across our sites and referral network. It includes 10 consensus statements that emphasize a harms reduction approach including patient and clinician education, assessment of patients for risks, counterindications, and monitoring for functional outcomes. 2) development of a pharmacist run monthly live and virtual education sessions to support patients in making informed choices about cannabis’ role in pain. From an evaluation of these sessions, we will share preliminary findings on how our patients use cannabis for pain and lessons learned around knowledge translation. We will discuss the factors that affect knowledge acquisition and describe where and how our patients are seeking information and what systemic barriers they encounter in accessing trustworthy information on cannabis’ use in pain. These findings will guide future program development and provide an indication of areas in which we, as healthcare providers, can further support our patients to use cannabis safely.

**Speaker 3**: Dr. Hance Clarke, MD, PhD, FRCPC, Department of Anesthesiology and Pain Medicine-University Health Network, Toronto Academic Pain Medicine Institute, Toronto, Ontario, Canada. Hance.Clarke@uhn.ca

**Speaker 3 Abstract Title**: The Future of Medical Cannabis and research initiatives to unlock the Pain Puzzle

Speaker 3 Abstract: In the context of cannabis legalization, 40% of Canadians report having used cannabis in their lifetime and 10% are n ow using plant based products routinely, indicating the use of cannabis for medical purposes is growing (3) However, no high-quality clinical trials of cannabis for common painful conditions such as osteoarthritis and fibromyalgia have been conducted, leaving physicians struggling to guide and properly inform patients regarding symptom relief. Findings from clinical trials of cannabis for other painful conditions have been variable, perhaps due to suboptimal cannabis products and failure to consider important patient characteristics. The future of medical cannabis integration into the global pain population is imminent. This presentation will discuss global trends in the evolving cannabis industry and results from two observational studies will be presented 1) Over 600 consecutive patients presenting to orthopaedic surgeons with a primary complaint of chronic MSK pain 2) 1000 patients presenting to Cannabis Clinics in Ontario were followed longitudinally. Data from the above work demonstrate that 1 in 5 patients presenting for a hip/knee operation are using cannabis for their OA. Longitudinal analyses from the second database highlight improvements in pain intensity with plant based cannabis products, however also uncover a sex bias in response to pain related outcomes. Finally, we will provide a snapshot into CIHR funded research looking at cannabis as a potential tool for OA management and other ongoing basic science & clinical cannabis studies that will aim to help guide the average Canadian physician and patient in the years ahead.

**Learning Objective 1**: Describe the patient journey

**Learning Objective 2**: Describe the programmatic initiatives developed to address patient knowledge gaps around cannabis use for chronic pain.

**Learning Objective 3**: Contextualize medical cannabis use in the Canadian population and provide some guidance for health care workers interested in the introduction of cannabis into patient care and provide a framework to guide future work

# Abstract: Knowledge translation interventions across the lifespan: Strategies for promoting uptake of evidence-based pain interventions from infancy to older adulthood

**Authors:** Thomas Hadjistavropoulos, Marsha Campbell-Yeo, Nicole E. MacKenzie

**Affiliations:** a Centre on Aging and Health and Department of Psychology, University of Regina, Regina, Saskatchewan, Canada; b School of Nursing, Faculty of Health, Departments of Pediatrics, Psychology and Neuroscience, Dalhousie University, Halifax, Nova Scotia, Canada; c Psychology and Neuroscience, Dalhousie University, Halifax, Nova Scotia, Canada

## Abstract

**Symposium Chair**: Thomas Hadjistavropoulos, Ph.D., FCAHS, Centre on Aging and Health and Department of Psychology, University of Regina, Regina, Saskatchewan, Thomas.Hadjistavropoulos@uregina.ca, @URHealthPsycLab

**Symposium Abstract**: Scientific knowledge in the field of pain is well-established and continues to grow exponentially. In spite of this, there is a significant knowledge-to-action gap that prevents the implementation of this knowledge into practice. Therefore, knowledge translation (KT) interventions are critical in promoting the uptake of best practices for pain management across the lifespan. The objectives of this symposium are to understand the role of KT interventions to improve the uptake of evidence-based pain management strategies across the lifespan. This symposium will provide an overview of KT interventions for pain in three different populations. The first presentation will illustrate the utility of a KT video intervention for promoting the use of evidence-based pain management strategies for infants to manage pain from medical procedures. The second will highlight childhood and adolescence and will present factors that influence use of a parent-directed KT intervention (published in Canadian parenting magazine) on strategies for children’s vaccination pain management. The third will present and summarize the implementation of a KT social media intervention to raise awareness about pain in patients with dementia who are not able to advocate for themselves. The importance of tailoring KT interventions to the needs of different knowledge users across the lifespan and challenges associated with evaluating impact of these interventions will be highlighted. The various KT interventions discussed during these sessions will be disseminated during the symposium to demonstrate possibilities for the development of these interventions for various knowledge users.

**Speaker 1**: Marsha Campbell-Yeo, PhD NNP-BC RN, Dalhousie University, Professor, School of Nursing, Faculty of Health, Departments of Pediatrics, Psychology and Neuroscience, Halifax, Nova Scotia, Canada, marsha.campbell-yeo, @DrMCampbellYeo

**Speaker 1 Abstract Title**: Targeting infant pain: Using e-health knowledge translation strategies to promote engagement in pain management behaviours for infants

**Speaker 1 Abstract**: No parent wants to see their baby in pain. Yet, despite known effective treatments to reduce pain associated with commonly performed procedures in early life, pain in infants still goes widely untreated. Not only does this evidence to practice gap reflect a lack of knowledge uptake and behavioral change across care providers, the majority of parents are unaware of the powerful role they can play in reducing their baby’s pain. One way to more fully engage care providers and families is through the use of innovative e-health technologies and social media knowledge translation (KT) strategies. Results of recent reviews examining the current status, quality and effectiveness of available content for parent targeted infant pain related resources provided via the Internet and mobile applications will be discussed. Moreover, preliminary findings regarding the development, implementation, feasibility and user acceptance of a parent targeted interactive e-Health platform will be reviewed, with particular focus on parental engagement in pain reducing behaviors while in the NICU. Findings from parent interviews, self-report through daily diaries and online analytics will be presented. To conclude, a brief overview of m-Health projects using alternative forms of e-Health technologies to target parents of healthy and at-risk newborns in both developed and developing countries from a scalability perspective across other populations and contexts will be examined.

**Speaker 2**: Nicole E. MacKenzie, MEd, Dalhousie University, Psychology and Neuroscience, Halifax, Nova Scotia, Canada, nmackenzie@dal.ca, @NMacKenzie_

**Speaker 2 Abstract Title**: Bridging the gap: Understanding parents’ use of a knowledge translation intervention for children’s vaccination pain management

**Speaker 2 Abstract**: Pain management is a primary concern of parents during vaccination procedures, and while many parents consider children’s pain management a priority, few are aware of the evidence-based practices in this area. Although knowledge translation (KT) interventions exist for pediatric pain, little is known about what parents think about these types of interventions or how they use this information in the context of a vaccination procedure. This presentation will describe factors related to parents’ planned and actual use of a KT intervention on vaccination pain management strategies and explore parents’ perceptions of the intervention itself, as assessed through an exploratory sequential, mixed-methods study. The quantitative survey component was theoretically informed by the Information Assessment Method for Parents (IAM-Parents). Survey findings of 128 parents highlighted the importance of creating KT interventions that are relevant to the context in which parents plan to use the information, in order to promote confidence in their continued use of strategies. Results from qualitative descriptive interviews with 20 parents further explored novel insight into the factors associated with parents’ present and future use of the KT intervention in a vaccination setting. This presentation will highlight the role of mixed-methods research in examining research questions regarding both KT and implementation science. Ultimately, these results will highlight the design considerations relevant to promoting the uptake of pain management strategies presented in KT interventions so that parents are well equipped to adhere to their child’s vaccination schedule, something which is more important than ever during the time of COVID-19.

**Speaker 3**: Thomas Hadjistavropoulos, Ph.D., FCAHS, Centre on Aging and Health and Department of Psychology, University of Regina, Regina, Saskatchewan, Thomas.Hadjistavropoulos@uregina.ca, @URHealthPsycLab

**Speaker 3 Abstract Title**: Inadequacies in Knowledge Translation and Ways of Moving Forward: A Focus on Pain in Dementia

**Speaker 3 Abstract**: There are significant delays in the translation of clinically important findings into widespread application. In addition, knowledge translation (KT) studies often focus solely on ability to increase health care staff knowledge and related satisfaction and do not tend to examine the impact of KT on patient outcomes. To overcome the long gap between practice and application non-traditional approaches to KT may be necessary. We will describe the outcomes of a large scale systematic effort to use social media in the dissemination of clinically important information on pain assessment in dementia. Moreover, ways of overcoming inadequacies in the evaluation and outcomes of published KT efforts will be discussed. Evidence suggests that traditional health care staff education (i.e., a form of KT), in and of itself, may be insufficient in inducing sustainable clinical change in the pain care of seniors with severe dementia. Work conducted by our group underscores the importance of: (a) combining continuing health care education with systematic implementation plans; (b) increased focus on patient outcomes in the evaluation of KT efforts; and (c) using non-traditional methods (i.e., social media) for disseminating knowledge to health care staff and stake holders.

**Learning Objective 1**: To understand the goals of knowledge translation and its significance in improving service provision and managing pain.

**Learning Objective 2**: To explore the importance of knowledge translation interventions to the needs of different knowledge users across the lifespan.

**Learning Objective 3**: To understand the challenges in evaluating KT interventions and provide strategies for addressing limitations around relevance, utility, and dissemination.

# Abstract: Understanding pain as bodily threat: new perspectives to unite psychological assessment, human neuroscience, and behavioral treatment.

**Authors:** Lauren Heathcote, Lydia Tam, Javeria Ali Hashmi, Johan Vlaeyen

**Affiliations:** a Department of Anesthesiology, Perioperative, and Pain Medicine, Stanford University School of Medicine, CA, USA; b Stanford University School of Medicine, CA, USA; c Department of Anesthesia, Pain Management, and Perioperative Medicine, Dalhousie University, Halifax, Nova Scotia, Canada; d KU Leuven, Research Group Health Psychology, KU Leuven, Leuven, Belgium

## Abstract

**Symposium Co-Chairs: **1) Lauren Heathcote, PhD (see info below) and 2) Lydia Tam, Patient Partner (BS, Stanford University School of Medicine, California, USA, lydiatam@stanford.edu).

**Symposium Abstract**: From an evolutionary perspective, pain functions to signal actual or potential bodily threat and thus to promote protective behaviors. The perception of pain as a signal of bodily threat has been relatively well-considered in some research areas (e.g., learning psychology), leading to novel treatment innovations (e.g., exposure). However, there remains a need to unite diverse clinical disciplines in order to create a shared understanding of this perspective that crosses mind, brain, and behavior. In this symposium, we will share new scientific innovations that seek to understand pain-threat interactions from the perspective of psychological assessment, fMRI human neuroimaging, and behavioral treatment, all within the context of the lived experience of a patient partner. Lydia Tam, a patient partner, will first describe her lived experience of pain and symptom perception within the threatening context of childhood cancer survivorship. Second, Dr. Lauren Heathcote will present the development and initial validation of a new psychological assessment tool, the Bodily Threat Inventory (BTI). Third, Dr. Javeria Ali Hashmi will present the neural and psychological underpinnings of why the threat of experiencing pain has a biasing effect on pain perception. Finally, Dr. Johan Vlaeyen will share recent findings regarding the behavioral approach to chronic pain, and how creating prediction errors can reduce dysfunctional avoidance behavior. The chairs and speaker team will then facilitate an active discussion on how to unite research on pain-threat interactions across clinical disciplines, particularly integrating the patient perspective, psychological assessment, and human neuroscience into the development of targeted and mechanistically precise treatments.

**Speaker 1**: Lauren Heathcote, PhD, Stanford University School of Medicine, Department of Anesthesiology, Perioperative, and Pain Medicine, California, USA, @lcheath@stanford.edu, @LCHeathcote

**Speaker 1 Abstract Title**: The Bodily Threat Inventory (BTI): a new self-report tool to assess the perception of pain and other somatic symptoms as signals of bodily threat

**Speaker 1 Abstract**: Somatic sensations, including pain, have evolved to act as signals of bodily threat and thus to promote behaviors that protect the body. At the conscious level, this likely reflects the interpretation of pain as indicating that something is wrong with the body (e.g., illness, injury). Yet, we know very little about how individuals attend to and interpret pain as a signal that something is wrong with their body, particularly within the context of complex and uncertain health conditions (e.g., chronic pain, cancer survivorship). A self-report measure that assesses how individuals perceive pain, and other somatic sensations, as indicating bodily threat is necessary for our psychological assessment toolkit and will align symptom science with the function of those symptoms. In this talk, Dr. Heathcote will present data on the development and preliminary validation of the Bodily Threat Inventory (BTI). She will show across adult (N>250) and pediatric (N>80) illness populations that the BTI has acceptable internal consistency and can reliably capture how individuals attend to, interpret, and worry about symptoms as signals of bodily threat. She will show that the BTI covaries with pain and health-related outcomes in adults and youth with a history of chronic illness, thus indicating strong criterion validity. The BTI will be a useful addition to our psychological assessment toolkit to better understand how individuals perceive pain as a signal of bodily threat within a variety of acute and chronic illnesses, thus better aligning pain science with the evolved function of pain.

**Speaker 2**: Javeria Ali Hashmi, PhD, Dalhousie University, Department of Anesthesia, Pain Management, and Perioperative Medicine, Halifax, Nova Scotia, Canada, @Javeria.hashmi@dal.ca, @netphys1

**Speaker 2 Abstract Title**: Threat prediction and its role in pain modulation

**Speaker 2 Abstract** (250 words or less): Chronic pain is one of the least understood clinical problems of our time. It is increasingly clear that nociceptive pathophysiology only tells us part of the story, and that brain mechanisms associated with expectations contribute to the experience of pain. Dr. Hashmi and her team have recently demonstrated that the expectation of receiving less pain reduces perceived pain intensity by up to 70%. Thus, the brain needs to be seen as an active participant in the generation of pain perceptions, so that we can begin to understand the fundamental neural mechanisms by which chronic pain arises. To date, very little is known about how the brain mediates the effects of top-down processes on pain perception. In this talk, Dr. Hashmi will show the brain’s structural and functional connections which explain the susceptibility for perceptions to be biased towards expectations instead of relying on bottom-up sensory evidence in some individuals. In addition, we know that the PAG (periaqueductal gray area) is engaged during pain modulation; however, how threat processing and pain modulation interface within distinct PAG columns is unclear. Triangulated findings suggest that the dorsal- lateral PAG may be involved in detecting salient threats and the ventrolateral PAG in contextualization and semantic appraisal of threats. Furthermore, Dr. Hashmi will discuss the patterns of whole brain connectivity and PAG circuits that explain why pain catastrophizing and mindful-awareness tend to vary with top-down threat bias. Identifying the neurocognitive predictors related to increased top-down threat bias should aid patient group stratification and tailored treatment-plan development.

**Speaker 3**: Johan Vlaeyen, PhD, KU Leuven, Research Group Health Psychology, KU Leuven, Leuven, Belgium, johannes.vlaeyen@kuleuven.be , @JohannesVlaeyen

**Speaker 3 Abstract Title**: Creating prediction errors in order to reduce pain avoidance

**Speaker 3 Abstract**: Beyond being a sensory experience, chronic pain is also part of a motivational system that alarms, directs, and energizes behavioral actions to minimize impending bodily harm. This flexible system enables one learning to predict, prevent and control harmful events in a continuously changing environment. One of the most frequent pain actions is preventing harm by avoiding stimuli that have been associated with pain, either by direct experience, observation or verbal instruction. Avoidance is adaptive in the presence of actual or potential harm, but it becomes dysfunctional in the absence of such harm, or when costs of avoiding are higher than the benefits. Although avoidance often competes with other behaviors that serve non-pain life goals, the extinction of avoidance is an arduous and fragile process. Repeated prediction “errors” are needed for extinction to occur, which can be created using exposure to the predictive cues in combination with response prevention. In this talk Dr. Vlaeyen will present recent data showing how exposure-led prediction errors can lead to behavioral changes in adults and youth with chronic pain, and how these help the individual to shift behavioral priority from pain avoidance towards valued life goals. He will also present challenges for future research in this area.

**Learning Objective 1**: Recognize how pain functions to signal bodily threat and to promote protective behaviors, thus understanding pain from an evolutionary perspective.

**Learning Objective 2**: Identify self-report tools and brain imaging approaches that help us to understand how pain is perceived as a signal of bodily threat and how this perception influences pain perception in a top-down manner.

**Learning Objective 3**: Describe a novel treatment approach that showcases how studying pain- threat interactions across clinical disciplines can lead to pain treatment innovation.

# Abstract: People who Live with Chronic Pain - Efforts in Research and Beyond

**Authors:** Richard Hovey, Jennifer Daly-Cyr, Therese Lane, Jacques Laliberté

**Affiliations:** a McGill University, Dentistry, Montreal, Quebec, Canada; b McMaster University Medical Centre, Hamilton, Ontario, Canada

## Abstract

**Symposium Chair**: Richard Hovey, MA, PhD, McGill University, Dentistry, Montreal, Quebec, Canada, Richard.hovey@mcgill.ca

**Symposium Abstract**: People who live with chronic pain are becoming more involved in the entire research process, from idea generation and priority-setting, right through to dissemination of research results / findings. While this has been happening organically in Canada, the Strategy for Patient-Oriented Research (SPOR) Chronic Pain Network has provided an organized catalyst for this approach since it was funded by CIHR in 2016. This symposium represents the CPN’s continuum of research engagement through the perspectives and personal experiences of four individuals who live with chronic pain and who are engaged with the Network. Moderated by an individual who lives with chronic pain and who is both a patient perspective consultant and researcher, other individuals who live with chronic pain will also share how they are: pushing the research agenda in the area of undiagnosed chronic pain; working with researchers, trainees and clinicians to develop training and best practice materials to guide patient engagement in chronic pain research and knowledge dissemination; and, moving these efforts in to the policy realm through engagement with Health Canada and involvement in the Canadian Pain Task Force. From the perspectives of those who live with chronic pain, the symposium intention is to share these CPN experiences as well as help audience members understand what resources are available to engage patients as perspective partners in their own work – in research and beyond. Learning from patient experience by researchers and clinicians will be discussed with recommendations for future engagement as a community of researchers.

**Speaker 1**: Jennifer Daly-Cyr, BComm, MA, Patient Perspective Partner Chronic Pain Network (CPN), McMaster University Medical Centre, 1280 Main St W, Hamilton, Ontario, Canada, L8S 4L8, jen.dalycyr@gmail.com

**Speaker 1 Abstract Title**: Undiagnosed: Is it Rare or is it an Epidemic? One Patient’s Quest for Answers and Research

**Speaker 1 Abstract**: An estimated 1 in 5 - or more than 6 million Canadians - are living with a chronic pain condition; what is not known is how many of these people are living with a chronic pain condition that is undiagnosed. According to the Undiagnosed Disease Network, “an undiagnosed disease is a medical condition without a known cause despite a lot of evaluation.” The speaker is one of the unknown number of people living with chronic pain due to an undiagnosed condition.

According to the Rare and Undiagnosed Organization in the US, “1 in 10 Americans struggle with rare or undiagnosed conditions”? However, little is research is published on undiagnosed chronic pain in Canada and little research is being conducted in this area.

This talk will help raise awareness and understanding about the plight of a constant state of unknown based on one individual’s lived experience. Further, her engagement as a patient perspective consultant with the CPN has been motivated by this experience, her findings that little research is being done in the area of undiagnosed chronic pain, and leads her to continuously advocate for undiagnosed chronic pain to be an area of research that is of critical importance to many Canadians.

**Speaker 2**: Therese Lane, Patient Perspective Partner Chronic Pain Network (CPN), McMaster University Medical Centre, 1280 Main St W, Hamilton, Ontario, Canada, L8S 4L8, therese.lane@icloud.com

**Speaker 2 Abstract Title**: Engaging People with Lived Experience – You Can Do It Too

**Speaker 2 Abstract**: The Chronic Pain Network has engaged patients as perspective partners throughout the research activities of the Network since its official start in 2016, including as research partners on specific projects and as ambassadors to help researchers and trainees in the Network engage other patient perspective partners. This talk will explain how the CPN’s Patient Engagement Committee, comprised of people living with chronic pain and researchers, has: been instrumental in providing training opportunities around patient perspective engagement in research; created a webinar series for trainees and researchers to talk about their work and seek input from people who live with chronic pain; and, also created resources where it has felt that gaps existed in the Network. An overview of the Patient Engagement Committees activities will be provided, including how people with chronic pain have been instrumental carrying them out. Audience members will also be introduced to and shown how they can use CPN’s resources related to patient engagement in their own research efforts.

**Speaker 3**: Jacques Laliberté, BCom, Past President and a Founding Director, Association québécoise de la douleur chronique (AQDC); Patient Perspective Partner CPN and Co-Chair, Executive Committee; Member of the Canadian Pain Task Force (CPTF), McMaster University Medical Centre, 1280 Main St W, Hamilton, Ontario, Canada, L8S 4L8,

**Speaker 3 Abstract Title**: People Living with Chronic Pain – Beyond Research to Policy

**Speaker 3 Abstract**: In response to the lack of a national organization that represents people who live with chronic pain, the Chronic Pain Network has provided policymakers and individuals in government an organizational conduit to people who live with chronic pain. While the Chronic Pain Network is a research network, a number of its patient perspective consultants are also involved in advocacy activities and are interested in the use of research evidence to inform policy. This session will share how the Chronic Pain Network developed a relationship with Health Canada specifically with respect to engaging people who live with chronic pain for various purposes. Initially Health Canada sought the opinions of people who live with chronic pain with respect to its website and a national advertising campaign and videos about opioid use and policy. From then, individuals who live with chronic pain who are involved in CPN have also been asked to participate in a roundtable with the Federal Minister of Health at the Health Canada Opioid Summit (September 2018), attend and contribute to presentations and conversations at the Opioid Summit, and also participate in the Canadian Pain Task Force and External Advisory Panel on Chronic Pain. These examples demonstrate how CPN as a research network has also become engaged in informing health policy.

**Learning Objective 1**: Understand how people who live with chronic pain are engaged in research and beyond in Canada through the SPOR Chronic Pain Network.

**Learning Objective 2**: Understand the types of activities and tools the Chronic Pain Network has created that are available for the research community to learn from and use in their own efforts to engage people who live with chronic pain in the research process.

**Learning Objective 3**: Understand how the experiences of people who live with chronic pain have motivated them to become involved in the Chronic Pain Network as well as activities that have resulted from that engagement more broadly.

# Abstract: Innovations in post-surgical pain management across lifespan: Patient partner, clinical, and research perspectives

**Authors:** Joel Katz, Janice Sumpton, Maria Pavlova, Hance Clarke

**Affiliations:** a York University, Faculty of Health, Department of Psychology, Toronto, Ontario, Canada; b Patient Partner, London, Ontario, Canada; c University of Calgary, Department of Psychology, Calgary, Alberta, Canada; d Department of Anesthesiology and Pain Medicine-University Health Network, Toronto Academic Pain Medicine Institute, Toronto, Ontario, Canada

## Abstract

**Symposium Chair**: Dr. Joel Katz, PhD, York University, Faculty of Health, Department of Psychology, Toronto, Ontario, Canada. jkatz@yorku.ca

**Symposium Abstract**: Millions of children and adults undergo surgeries annually. Post-surgical pain is often inadequately managed, distressing, and may result in traumatic memories of the surgical experience. Importantly, up to 25% of patients are at risk for developing chronic post-surgical pain (CPSP), contributing to the rising epidemic of chronic pain. The research community, clinicians, parents of children undergoing surgeries, and adult patients have indicated an urgent need for non-pharmacological pain management to alleviate post-surgical pain and the associated suffering, as well as to prevent the pain from becoming chronic. In the proposed symposium, our patient partner will share her lived experience of a childhood surgery that she vividly remembers and that is tied to her chronic pain condition that persists to this day. She will discuss her experiences of this surgery and post-surgical pain management and will offer a unique perspective on how it shaped her future pain experiences. Ms. Pavlova will present new findings from a randomized controlled trial of a novel parent-led memory-reframing intervention aimed at positively altering young children’s memories of post-surgical pain. Dr. Clarke will present new data from the Transitional Pain Service (TPS), a specialized peri- and post-operative pain management program. Specifically, Dr. Clarke will discuss how the integration of the Manage My Pain app into the TPS influenced patient engagement and patient outcomes. The panel includes an interdisciplinary group of clinical researchers and a patient partner applying a patient-centred and developmentally-informed lens to the impact of post-surgical pain and new and innovative avenues in its management.

**Speaker 1**: Janice Sumpton, Patient Partner, London, Ontario, Canada, janice.sumpton@gmail.com, @JaniceSumpton

**Speaker 1 Abstract Title**: The long-term impact of childhood post-surgical pain: Lived experience.

**Speaker 1 Abstract**: Ms. Sumpton will share her lived experience and pain journey from birth to adulthood, and specifically the influence her childhood surgery had on her chronic pain condition that persists to this day. Ms. Sumpton was born prematurely when it was widely believed that newborns did not feel pain. She spent the first six weeks of life at the hospital, where she underwent numerous painful procedures to address peri- and post-natal complications. At an early age she experienced pain that was initially disbelieved by her parents and later diagnosed as a leg fracture. Different ongoing pain had to be surgically addressed at ten years of age. Pre-operative trauma (i.e., being held down) and post-operative pain were suffered in silence. Limb pain persisted into Ms. Sumpton’s young adult years. At the age of 38 years, she was diagnosed with neuropathic back pain. From ages 38-60 years Ms. Sumpton required ten major surgeries. Each surgery added the burden of acute post-surgical pain to chronic pain (neuropathic pain and fibromyalgia). Ms. Sumpton will share how numerous medical procedures, specialist appointments, hospital visits, and persistent pain affected her life, work, and family. She will elaborate on resiliency factors that helped her to persevere and thrive in the presence of chronic pain and trauma (e.g., supportive family, optimism, involvement in patient advocacy groups). Ms. Sumpton will discuss the impact of childhood pain, and memories of it, on her life and ways in which pain both could and should be managed from a patient perspective.

**Speaker 2**: Maria Pavlova, MSc, University of Calgary, Department of Psychology, Calgary, Alberta, Canada, mpavlova@ucalgary.ca, @mariavpavlova

**Speaker 2 Abstract Title**: Reframe the Pain: A Parent-Led Intervention to Alter Children’s Memories for Pain

**Speaker 2 Abstract**: Pain is common in childhood and influences children long after the painful experience ends. Children’s memories for pain robustly predict future pain experiences. Negatively-biased pain memories (i.e., recalling higher levels of pain as compared to initial/earlier pain reports) in childhood are associated with future pain, distress, and avoidance of medical care into adulthood. Children’s memories for pain are highly malleable, yet there have only been three studies examining memory-reframing techniques and all in the context of needles. These memory-reframing interventions were efficacious in reducing negative biases in pain memories and distress at future *needle* procedures (Noel et al., 2018); however, recall of *post-surgical* pain that can be distressing and lead to persistent pain problems have not yet been targeted. Further, existing memory-reframing interventions excluded parents, who play a critical role in the formation of children’s memories and who are underutilized intervention agents. Empirical research demonstrated that children’s pain memories are influenced by how parents reminisce with children about the surgical experience. Parents who reminisced in more elaborative ways, discussed more positive emotions, and used less pain words had children who developed less negatively-biased pain memories (Noel et al., 2019). Research has not yet examined if memory-reframing techniques used by parents may *change* children’s recall of post-surgical pain. Ms. Pavlova will present results of the first RCT examining the efficacy of a novel parent-led memory-reframing intervention aimed at reducing negative biases in children’s pain memories following surgery and will discuss new avenues in pediatric post-surgical pain management to improve long-term outcomes.

**Speaker 3**: Dr. Hance Clarke, MD, PhD, FRCPC, Department of Anesthesiology and Pain Medicine-University Health Network, Toronto Academic Pain Medicine Institute, Toronto, Ontario, Canada. Hance.Clarke@uhn.ca, @drhaclarke

**Speaker 3 Abstract Title**: Novel Interventions in Transitional Pain Care

**Speaker 3 Abstract**: The Transitional Pain Service (TPS) has developed a multidisciplinary framework which enables targeted, mechanism-based treatment for patients who are at risk of chronic post-surgical pain and disability following surgery. The TPS provides specialized care perioperatively and after hospital in-person, by telephone, or via online video calling (e.g., telehealth private link) and via the integration of mobile technology. The crossover of mobile technology and healthcare is becoming increasingly popular, with over 318,000 health apps available worldwide in 2017 – nearly double the number of apps available in 2015 – and more than 200 apps being added each day. This has great potential in the treatment of chronic pain, with apps already providing support as electronic diaries, pain assessment, and activity trackers for patients. However only five apps reported the involvement of a health care provider in their development (Sundararaman et al., 2017). Manage My Pain(MMP) is one of these apps, and we have integrated the use of it, along with its remote monitoring capabilities, into our TPS population. This data will be presented during this symposium. We evaluated patient engagement with MMP and differentiated between high- and low-engagement users. We will present engagement data both within the TPS and for remote users. In the summer of 2020 during COVID-19, MMP has become the primary database of the service and is integrated into clinical flow. We will present engagement data and present data evaluating the potential impact of MMP in terms of clinical endpoints such as on pain severity, pain catastrophizing and opioid weaning.

**Learning Objective 1**: Provide a patient partner perspective of pain, trauma, and pain memories and their profound impact across lifespan.

**Learning Objective 2**: Examine the efficacy of parent-led memory-reframing intervention aimed at positively altering children’s memory for post-surgical pain.

**Learning Objective 3**: Describe the barriers and facilitators to implementing mobile technology into the Transitional Pain Service and present patient related outcomes.

## References

[1] Noel, M., McMurtry, C. M., Pavlova, M., & Taddio, A. (2018). Brief report: A systematic review and meta-analysis of pediatric memory reframing interventions for needle procedures. *Pain Practice*, 18(1), 123–129.2829616410.1111/papr.12572

[2] Noel, M., Pavlova, M., Lund, T., Jordan, A., Chorney, J., Rasic, N., Brookes, J., Hoy, M., Yunker, W., & Graham, S. (2019). The role of narrative in the development of children’s pain memories: Influences of father- and mother-child reminiscing on children’s recall of pain. *PAIN*.10.1097/j.pain.000000000000156531335654

[3] Sundararaman, L. V., Edwards, R. R., Ross, E. L., & Jamison, R. N. (2017). Integration of mobile health technology in the treatment of chronic pain: A critical review. *Regional anesthesia and pain medicine*, 42(4), 488–498.2857043610.1097/AAP.0000000000000621

# Abstract: COVID-19, human nociceptors, and pain

**Authors:** Rajesh Khanna, Theodore Price

**Affiliations:** a University of Arizona, Department of Pharmacology, Tucson, AZ, USA; b University of Texas at Dallas, Neuroscience, Dallas, TX, USA

## Abstract

**Symposium Chair:** Rajesh Khanna, PhD, University of Arizona, Department of Pharmacology, Tucson, Arizona, USA marsha.rkhanna@arizona.edu @KhannaLabUA

**Symposium Abstract**: Severe acute respiratory syndrome coronavirus 2 (SARS-CoV-2) is the causative agent of COVID-19, a coronavirus disease that, as of November 10, has infected more than 50 million people and caused over 1,200,000 deaths worldwide and paralyzed global economies. The pandemic continues unabated and certain aspects of the disease continue to baffle clinicians and researchers. It has been suggested that transmission of the SARS-CoV-2 by asymptomatic or mildly symptomatic individuals could be responsible for up to half of the spread, which is why the virus has been so difficult to contain. In this symposia, we discuss: (1) the molecular gateways used by the virus to enter the nervous system, particularly the nociceptors; (2) the cytokine storm as a potential cause of the profound and long-lasting effect on COVID-19; (3) the potentially analgesic effect of the SARS-CoV-2’s spike protein results in a reduced pain response during infections, thus making this virus even more insidious; (4) testimonials from chronic pain patients who report transient pain loss during COVID-19. Prior to the ‘surprise’ emergence of the COVID-19 pandemic in December of 2019, the United States and parts of the World were mired by the opioid epidemic. Thus, the findings presented in this symposium are relevant to two current global health crises as emerging data suggest that the COVID-19 pandemic is likely to compound the opioid epidemic. Overall, the symposia will inform how COVID-19 may influence long-term pain outcomes.

**Speaker 1**: Theodore Price, PhD, University of Texas at Dallas, Neuroscience, Dallas TX USA Theodore.price@utdallas.edu @UTDpainlab

**Speaker 1 Abstract Title**: The effect of COVID-19 on human nociceptors

**Speaker 1 Abstract**: COVID-19 is caused by the SARS-CoV-2 virus which is known to infect cells through ACE2, using neuropilin receptors as an important infection cofactor. People that develop moderate to severe symptoms of COVID-19 experience dramatic changes in systemic cytokine levels that may cause what is referred to as “cytokine storm”. This appears to be an important cause of morbidity and mortality in the disease. Our work has focused on two questions: 1) Can SARS-CoV-2 infect human nociceptors; and 2) How might the cytokines found in COVID-19 influence human nociceptors. To answer the first question we have addressed expression of ACE2 and neuropilin 1 in human dorsal root ganglion (DRG) nociceptors. We find that both receptors are expressed by human nociceptors. We are now pursuing whether these neurons are infected in vitro. To address the second question, we have used computational approaches, which suggest that the cytokine storm in COVID-19 is likely to have a profound and long-lasting effect on human nociceptors. This work will be discussed in the context of how COVID-19 may influence long-term pain outcomes.

**Speaker 2: Rajesh Khanna**, PhD, University of Arizona, Department of Pharmacology, Tucson, Arizona, USA rkhanna@arizona.edu @KhannaLabUA

**Speaker 2 Abstract Title**: SARS-CoV-2 Spike protein co-opts VEGF-A/Neuropilin-1 receptor signaling to induce analgesia

**Speaker 2 Abstract**: Global spread of severe acute respiratory syndrome coronavirus 2 (SARS-CoV-2) continues unabated. Binding of SARS-CoV-2’s Spike protein to host angiotensin converting enzyme 2 triggers viral entry, but other proteins may participate, including neuropilin-1 receptor (NRP-1). As both Spike protein and vascular endothelial growth factor-A (VEGF-A) – a pro-nociceptive and angiogenic factor, bind NRP-1, we tested if Spike could block VEGF-A/NRP-1 signaling. VEGF-A–triggered sensory neuronal firing was blocked by Spike protein and NRP-1 inhibitor EG00229. Pro-nociceptive behaviors of VEGF-A were similarly blocked via suppression of spontaneous spinal synaptic activity and reduction of electrogenic currents in sensory neurons. Remarkably, preventing VEGF-A/NRP-1 signaling was antiallodynic in a neuropathic pain model. A ‘silencing’ of pain via subversion of VEGF-A/NRP-1 signaling may underlie increased disease transmission in asymptomatic individuals. Leveraging these findings, we report hits from a small molecule and natural product screen of nearly 0.5 million compounds targeting the VEGF-A binding site on NRP-1. These compounds target the neuropilin receptor 1 with the potential to interfere with SARS-CoV-2 virus entry and pain signaling.

**Speaker 3**: Chronic pain patients with COVID-19

**Speaker 3 Abstract Title**: Transient or permanent loss of pain in chronic patients with COVID-19

**Speaker 3 Abstract**: Real life experiences of patients with chronic pain whose pain levels dramatically changed when they contracted COVD-19:

Ms. Corbett is a chronic pain patient with last 22 years after an ankle injury at age 16. She was diagnosed with COVID on 10/28, symptoms starting on 10/27. About 3-4 days into the virus, she lost the ability to feel pain, and is pain free at the moment. She will share her life experience and journey with chronic pains and COVID-19.

Mr. Pretorius is chronic pain patient who lives with constant burning pain in his legs that wakes him up nightly at 3 or 4 A.M. “It feels like somebody is continuously pouring hot water over my legs,” Pretorius says. But that changed when he contracted COVID-19 in July at his job. “When I was sick with COVID, the pain was bearable. At some points, it felt like the pain was gone.” he says. Pretorius was able to sleep through the night for the first time since 2011. “I lived a better life when I was sick because the pain was gone,” despite having fatigue and debilitating headaches, he says. Now that Pretorius has recovered from COVID, his neuropathic pain has returned.

Ms. Sangha is a patient with nerve myopathy, fibromyalgia, migraines & arthritis. She lives in constant pain. She contracted COVID-19 and says that “I had no body pain. For months. It was absolute bliss”. She will share her life experience and journey with chronic pains due to spinal cord injury.

**Learning Objective 1**: What We Know So Far about How COVID Affects the Nervous System?

**Learning Objective 2**: Is there more than more molecular ‘gateway’ for SARS-CoV-2 viral entry?

**Learning Objective 3**: Do patients experience changes in their pain sensitivity during and after COVID-19?

# Abstract: Diverse phenotypes and functions of microglia in pain

**Authors:** Arkady Khoutorsky, Shannon Tansley, Simon Beggs, Michael Salter

**Affiliations:** a McGill University, Department of Anesthesia, Montreal, QC, Canada; b UCL Great Ormond Street Hospital Institute of Child Health, London, UK; c Hospital for Sick Children and University of Toronto, Toronto, ON, Canada

## Abstract

**Symposium Chair**: Arkady Khoutorsky, PhD, McGill University, Department of Anesthesia, Montreal, QC, Canada, arkady.khoutorsky@mcgill.ca

**Symposium Abstract**: The field of pain research has placed great emphasis on the mechanisms by which glial cells regulate pain. In this symposium, we will highlight new insights into the role of microglia in the development and maintenance of pain. The panelists will describe the diverse roles of microglia in the spinal cord by presenting microglia single-cell RNA sequencing data following nerve injury, and discussing microglia functions in shaping somatosensory/nociceptive circuits during development and in adulthood and the role of sex hormones and DNA methylation in these processes.

**Speaker 1**: Shannon Tansley, McGill University, Anesthesia, Montreal, Quebec, Canada, shannon.tansley@mcgill.ca

**Speaker 1 Abstract Title**: Characterization of microglia transcriptional states after nerve injury using single-cell RNA sequencing.

**Speaker 1 Abstract**: Microglia are resident immune cells in the central nervous system. Nerve injury induces an increase in the number of microglia in the spinal cord (microgliosis) and leads to profound changes in their phenotype. Silencing microglia using pharmacological (minocycline, Mac-1-saporin and CSF-1 inhibitors) and genetic (CX3CR1 deletion) approaches strongly alleviates the development of pain after nerve injury, suggesting a critical role of microglia in the pathophysiology of neuropathic pain. However, the mechanisms by which microglia promote the sensitization of spinal nociceptive circuits are not fully understood. Notably, microglia mediate nerve injury-induced hypersensitivity in male but not in female animals. Recent evidence suggests that microglia in the central nervous system are not uniform and consist of numerous sub-types, potentially playing diverse roles at different phases of pain development.

We have performed single cell RNA-sequencing on spinal cord microglia following nerve injury (spared nerve injury, SNI) in male and female mice at 3 days, 14 days, and 5 months after SNI. Our results demonstrate that microglia are a heterozygous population of cells, showing profound and in some cases sex-specific changes in gene expression after injury.

**Speaker 2**: Simon Beggs PhD, UCL Great Ormond Street Hospital Institute of Child Health, London, UK. s.beggs@ucl.ac.uk

**Speaker 2 Abstract Title**: Microglia control restructuring of spinal somatosensory circuits during normal development and after injury

**Speaker 2 Abstract**: Maturation of sensory systems is an activity-dependent process and requires modality specific input in early postnatal life. Spinal somatosensory circuits are functional at birth but undergo extensive postnatal reorganization, with refinement of afferent input and maturation of inhibitory circuits. Peripheral tissue injury during this period of maturation results in long-term changes in local and whole-body pain sensitivity, which can be prevented by pharmacological inhibition of spinal microglial activity at the time of injury. Microglia are known to refine neural circuits through selective strengthening or elimination of synapses in normal brain development and the activity of microglia in refining spinal somatosensory/nociceptive circuits during normal postnatal development through pruning will be described. Tissue injury during this period disrupts microglial pruning, thus altering circuit refinement and subsequent pain behavior. Neonatal disruption of microglial phagocytosis by both pharmacological methods and using a mouse genetic approach reveal changes in spinal somatosensory processing in adult animals using *in-vivo* electrophysiology, indicating microglial pruning is critical for the development of dorsal horn sensory function. These findings suggest a microglia-mediated refinement of afferent projections in the dorsal horn through phagocytosis and dysregulation of that process after early postnatal injury, which may underly subsequent pain sensitization in later life.

**Speaker 3**: Michael Salter MD PhD, Hospital for Sick Children and University of Toronto, Toronto, ON mike.salter@utoronto.ca

**Speaker 3 Abstract Title**: What’s new in sex, pain and microglia.

**Speaker 3 Abstract**: Neuron-microglial interactions are increasingly recognized as being key for physiological and pathological processes in the central nervous system. Microglia have been found to play a causal role in neuropathic pain behaviours resulting from peripheral nerve injury, and a core neuron-microglia-neuron signaling pathway has been elucidated. Within the dorsal horn, microglia suppress neuronal inhibition by a cascade involving increased expression and activation of microglial P2X4 receptors causing these cells to release brain derived neurotrophic factor (BDNF). BDNF acts on neuronal trkB receptors which leads to downregulation of the K+-Cl- co-transporter KCC2, resulting in a rise in intracellular Cl- concentration in dorsal horn nociceptive output neurons, transforming the response properties of these neurons. In addition to suppressing inhibition, peripheral nerve injury causes activity-dependent potentiation at dorsal horn glutamatergic synapses which enhances nociceptive transmission. BDNF mediates the enhancement of synaptic NMDAR responses through activation of TrkB and the Src-family kinase, Fyn. This core signaling pathway has been extensively characterized in studies using male mice. We have discovered, however, that microglia-to-neuron signaling is dispensable in female mice. Rather, pain hypersensitivity in female mice depends upon the adaptive immune system, likely upon T cells. The immune cell signaling in females converges on downregulation KCC2 and enhancement of NMDARs in dorsal horn neurons. Whether microglia or adaptive immune signaling mediates hypersensitivity depends upon sex hormones, and is linked to distinct epigenetic changes. Despite the profound differences in cellular mechanisms, pain hypersensitivity in female mice is as robust as that in male mice. Taking into consideration sex differences in the spinal immune-neuronal signaling has important implications ranging from diagnostics, to therapeutics, to prevention of chronic pain.

**Learning Objective 1**: Upon completion of this session, attendees will be aware of various transcriptional states of microglia in the spinal cord following nerve injury in male and female mice.

**Learning Objective 2**: Upon completion of this session, attendees will have an understanding of the normal developmental role of microglia in experience-dependent refinement of spinal neuronal connectivity.

**Learning Objective 3**: Upon completion of this session, attendees will be aware how sex hormones and DNA methylation may control spinal mechanisms of pain hypersensitivity after peripheral injury.

# Abstract: From Post-Operative Pain to Chronic Pain and Even In-Between: Understanding Psychological and Pharmacological Mechanisms of Pediatric Prolonged Pain States

**Authors:** Rebecca Pillai Riddell, Cheryl Chow, Deepa Kattail, Melanie Noel

## Abstract

**Symposium Chair:** Dr. Rebecca Pillai Riddell, C.Psych., York University, Department of Psychology, Toronto, Ontario, Canada, rpr@yorku.ca, @drbeccapr

**Symposia Abstract**: Up to 38% of youth suffer from chronic pain worldwide (Miro 2017). The frequent use of medical and related mental health services in managing pediatric pain often takes a toll on families, both physically and emotionally, and is also linked to high societal costs (Martin, 2018). This three-part series symposium describes multidisciplinary perspectives in pediatric pain contexts (i.e., acute, transitional and chronic). The focus of this talk will be on the mechanisms and models that likely contribute to pediatric pain, and identification of potentially modifiable risk factors that will lead to the development of successful prevention and intervention strategies. In the first talk, neuroscientist Chow discusses the current state of knowledge on the role of anxiety and its related states in pediatric postsurgical pain, and the potential interventions. The second speaker, physician Kattail, will opines on the importance of understanding the role of opioid pain management in transitional pain services for youths with chronic pain, using empirical data and case studies. Finally, clinical psychologist Noel, will review the intergenerational transmission of risk for pediatric chronic pain across generations, using new longitudinally epigenetic and behavioural data. Together, the current literature provides a current state of the art grounding in new directions of understanding pharmacological and psychological factors in pediatric pain beyond acute.

**Speaker 1**: Cheryl Chow, MSc. PhD., York University, Department of Psychology, Toronto, Ontario, Canada, chtchow@yorku.ca

**Speaker 1 Abstract Title**: The Role of Anxiety and its Related States in Predicting Pediatric Postsurgical Pain

**Speaker 1 Abstract**: Nearly 20% of children and adolescents have pain with disability 1 year after surgery and they experience poor sleep, school absence, and decreased activities. Negative clinical, psychological, and developmental effects include greater pain medication use, longer recovery, and fear of future medical care. Research has found psychological and family influences (i.e., child and parental anxiety) on pediatric chronic postsurgical pain (CPSP), but a better understanding of the role of perioperative anxiety and its related states in predicting pediatric postsurgical pain is needed. This talk will summarize the literature on child’s perioperative anxiety, and parental anxiety, in relation to acute postsurgical pain, CPSP, and pain trajectories. Other related psychological factors (i.e., anxiety sensitivity, catastrophizing, pain anxiety and fear of pain) in relation to pediatric acute and chronic postsurgical pain will also be discussed. These studies reveal the complex relationships and interconnectedness between child anxiety, parental anxiety, catastrophizing and postsurgical pain in children and adolescents undergoing surgeries. The findings may improve the current understanding of the causes of CPSP and highlight the gaps and need for further study.

**Speaker 2**: Deepa Kattail, MHS., MD., McMaster University, Department of Anesthesia, Hamilton, Ontario, Canada, kattaild@mcmaster.ca, @DeepaKattail

**Speaker 2 Abstract Title**: Interrupting the Trajectory of Pain: Transitional Pain Services To Prevent Acute Pain From Becoming Chronic

**Speaker 2 Abstract**: Chronic pain is a motivating factor for some patients to seek out surgical interventions. Often, these interventions are focused on a specific anatomical issue, without much consideration for the biopsychosocial factors that are influential in the experience of pain. Studies have shown that patients with underlying comorbidities, such as anxiety or higher levels of pain catastrophizing, have higher opioid consumption in the immediate postoperative period. What is the solution for a patient who has undergone surgery to relieve chronic pain, and instead is now experiencing even higher levels of pain? Opioids may be prescribed beyond the expected time of recovery and the patient may further deteriorate in terms of their pain status and mental health. Transitional pain services are a new model of care that bridges services in acute and chronic pain services and address situations where early intervention may prevent acute postoperative pain from escalating into a more debilitating chronic pain issue. Patients in this vulnerable phase are urgently triaged and introduced to a multidisciplinary approach to pain with a goal of weaning opioids and replacing it with evidence-based treatment including psychosocial support and physical therapy. Transitional pain services can also collaborate with surgeons and anesthesiologists for known chronic pain patients who are scheduled for surgery, in order to develop a customized perioperative pain strategy, including techniques to decrease pain sensitization and to also provide pre-operative pain coping strategies. Utilizing real case studies, the value of transitional pain services will be demonstrated in this talk.

**Speaker 3**: Melanie Noel, PhD, RPsych, University of Calgary, Department of Psychology, Calgary, Alberta, Canada, melanie.noel@ucalgary.ca, @MelanieNoel

**Speaker 3 Abstract Title**: Trauma and Pediatric Pain: An Intergenerational Problem

**Speaker 3 Abstract**: Early life trauma can lead to pain problems, which can persist for generations. Yet, how trauma ‘gets under the skin’ to ignite pain problems is unknown. Children with chronic pain and their parents experience trauma symptoms at much higher rates than non-pain populations and trauma is linked to worse pain and functioning. Nevertheless, trauma is rarely assessed or targeted in pain clinics. Conceptual models of mutual maintenance posit that neurobiological, cognitive-behavioral, and interpersonal factors drive this relationship, but this has not been empirically shown in longitudinal research. Dr. Noel will present new data from a cohort of treatment-seeking youth with chronic pain and their parents (N=200) integrating methods in brain-imaging, ecological momentary assessment, and activity monitoring demonstrating the roles of brain activation, sleep disturbances, and parent factors in the co-occurrence and maintenance of trauma (Adverse Childhood Experiences, PTSD) and pediatric chronic pain over time. Data demonstrating epigenetic and behavioural (parenting responses, sleep disturbances) mechanisms underlying the intergenerational transmission of risk for pediatric chronic pain across generations will be presented. This will parallel preclinical findings from rodent models. Overlapping epigenetic factors yielded from combining the animal and human models will be identified. The talk will end with a discussion of promising new treatment models to target underlying mechanisms and social factors to promote resilience and recovery.

**Learning Objective 1**: To summarize the literature on pediatric anxiety and parental anxiety, in relation to pediatric acute and chronic postsurgical pain.

**Learning Objective 2**: To examine the role of transitional pain services as a new model of care that bridges services in pediatric acute and chronic pain services.

**Learning Objective 3**: To longitudinally examine the link between trauma and pediatric chronic pain across generations.

# Abstract: Innovations in chronic pain management: individualized and targeted interventions

**Authors:** Nivez Rasic, Lauren E. Harrison, Vishal Varshney, Jillian Vinall Miller

**Affiliations:** a University of Calgary, Anesthesiology, Perioperative & Pain Medicine, Calgary, Alberta, Canada; b Stanford University School of Medicine, Department of Anesthesiology, Perioperative, and Pain Medicine, Stanford, CA, USA; c Providence Healthcare, Department of Anesthesia, Vancouver, BC, Canada

## Abstract

**Symposium Abstract**: Chronic pain affects adults more than heart disease, diabetes and cancer combined. It is also extremely debilitating, often impacting work, social, emotional and daily functioning. For many adults with chronic pain, their problems with pain began much earlier, in childhood. Two thirds of youth with chronic pain become adults with chronic pain. Moreover, youth with severe chronic pain are at an even greater risk for pain persisting into adulthood. It is imperative that we find ways to interrupt this pain trajectory and relieve suffering for these individuals. Three novel therapies, which demonstrate potential for modifying pain outcomes include: 1) graded in-vivo exposure treatment; 2) spinal neuromodulation; and 3) repetitive transcranial magnetic stimulation (rTMS). Dr. Lauren Harrison (Stanford University, Stanford, CA) will present findings from her graded exposure treatment (GET Living) in pediatric chronic pain. This is an individualized outpatient treatment program for youth with chronic pain that requires families to work jointly with therapists to progressively re-engage the patient in daily living activities. Dr. Vishal Varshney (Providence Healthcare, Vancouver, BC) will present his work on spinal stimulation in patients with chronic non-cancer pain. Finally, Dr. Jillian Miller (University of Calgary, Calgary, AB) will present her early findings exploring the benefit of adding rTMS to an intensive pain rehabilitation program (IPRP: three-week, interdisciplinary day treatment program) for youth with severe chronic pain. Each of these strategies aim to improve upon our current pain interventions, with the goal of providing patients with evidence-based, individualized care, the current gold standard for clinical intervention.

**Speaker 1**: Lauren E. Harrison, PhD Stanford University School of Medicine, Department of Anesthesiology, Perioperative, and Pain Medicine, Stanford, CA 94305. leharr@stanford.edu, Twitter: harrison_laur

**Speaker 1 Abstract Title**: GET Living! A functional approach to pediatric pain rehabilitation

**Speaker 1 Abstract**: Pain-related fear and avoidance are important factors effecting outcomes for children with chronic pain. Recently our group developed, trialed, and found effectiveness for an interdisciplinary, graded exposure intervention (GET Living) that targets pain-related fear avoidance in children with chronic pain. The purpose of the current study is to present GET Living and outline the clinical trial protocol. The intervention to be discussed is a graded in-vivo exposure treatment (GET Living) compared with typical pain management (TPM) in adolescents with chronic musculoskeletal pain. Data are collected at baseline, end of treatment, 3-month, and 6-month follow-up. Primary outcome is a pain-related fear avoidance. Secondary outcomes are functional disability, protective parent responses, and pain acceptance. Exploratory aims include biomechanics and daily physical activity. All participants and parents also complete a Daily Diary from baseline to end of treatment and at 3- and 6-month follow-up. GLM analyses conducted on feasibility trial data demonstrated significant decreases in avoidance (p<0.001), functional disability (p<0.001) and fear of pain (p=0.004). Of the 28 patients who completed the initial treatment, 25 patients showed clinically significant improvements in fear, avoidance, and/or functional disability, while 61% (n=17) showed clinically significant improvements in all three measures. Data from the feasibility study provided key data to inform the design of the subsequent RCT of an innovative outpatient treatment model integrating psychologists and physical therapists. Data will provide preliminary evidence of the effectiveness of GET Living on pain-related outcomes in children and adolescents with chronic pain.

**Speaker 2**: Vishal Varshney, MD FRCPC (Anesthe-siology and Pain Medicine), Providence Healthcare, Department of Anesthesia, Vancouver, BC, Canada, vvarshney@providencehealth.bc.ca, @VarshneyMD

**Speaker 2 Abstract Title**: Dorsal root ganglion stimulation for chronic pain – a new target for neuromodulation

**Speaker 2 Abstract**: The dorsal root ganglia (DRG) contain the cell bodies of primary sensory neurons, and have been implicated as a site of many pathophysiologic changes that occur in chronic pain. This structure is accessible in the spine via the epidural space, and is amenable to neuromodulation. The ability to apply electrical stimuli to this structure as a form of neuromodulation only became available in Canada in early 2020, yet evidence is emerging that shows stimulation of this structure to be superior to traditional spinal cord stimulation in patients with neuropathic pain. The Neuromodulation Program at St. Paul’s Hospital (Vancouver, BC, Canada) has implanted 4 patients with focal neuropathic pain with dorsal root ganglion stimulation devices, and will trial at least 8 more in advance of the 2021 Virtual Scientific Meeting. Pre- and post-intervention pain visual analog scales, pain interference as measured through the Brief Pain Inventory, pain catastrophizing measured through the Pain Catastrophizing Scale, functional improvement measured through the PROMIS-29 questionnaire, and opioid consumption (in morphine-equivalent daily doses) has and will be collected on all patients trialed and implanted. Preliminary data on implanted patients has demonstrated a 70% reduction in pain visual analog scale, 79% reduction in opioid consumption, and 40% improvement in pain catastrophizing scales. Dorsal root ganglion stimulation is a promising neuromodulation therapy with significant potential to improve patient quality of life in those with focal neuropathic pain.

**Speaker 3**: Jillian Vinall Miller, PhD, University of Calgary, Anesthesiology, Perioperative & Pain Medicine, Calgary, Alberta, Canada, jillian.miller1@ucalgary.ca, @JillVMiller

**Speaker 3 Abstract Title**: Can brain stimulation enhance outcomes associated with intensive rehabilitation for youth with chronic pain?

**Speaker 3 Abstract** (250 words or less): Severe chronic pain is defined as pain persisting for three months or more that significantly impacts daily functioning. It is highly prevalent, costly and persistent. In 2014, the Alberta Children’s Hospital established the first Canadian pediatric Intensive Pain Rehabilitation Program (IPRP; 3-week day-treatment program) to target youth with severe chronic pain and consequent functional disability who do not respond to outpatient pain therapies. Following IPRP, youth reported less pain interference (P=0.002), less functional disability (*P*<0.001), less anxiety (*P*=0.04), depressive (*P*<0.001) and PTSD symptoms (*P*=0.04), however, self-reported pain remained unchanged (*P*=0.29). In August 2016, we began to explore brain changes associated with the IPRP. We scanned a subset of youth at the start (baseline) and end (discharge) of IPRP (23 youth with 2 brain scans). From baseline to discharge we saw decreases in activity in the dorsolateral prefrontal cortex (DLPFC; *P*=0.02). The DLPFC is a well-known target for non-invasive brain stimulation. Repeated brain stimulation has been used to treat adults, but not youth with chronic pain. For the first time, we will use image-guided brain stimulation (37 minutes/day, 5 days/week) to enhance the brain changes observed with IPRP. We will examine whether three weeks of brain stimulation helps to reduce pain symptoms in youth. We will also compare pain, brain, and mental health outcomes to our historical program data. By adding brain stimulation to our pain intervention, we have the chance to target an area of the brain we know to be altered by chronic pain to improve outcomes.

**Learning Objective 1**: The use of interdisciplinary, graded exposure intervention for pain-related fear avoidance in children with chronic pain.

**Learning Objective 2**: Neuromodulation via dorsal root ganglion stimulation for patients with neuropathic pain.

**Learning Objective 3**: Benefit of adding repetitive transcranial magnetic stimulation to intensive pain rehabilitation for youth with severe chronic pain.

# Abstract: Mechanisms of pain learning and motivation: neurobiological mechanisms and computational models

**Authors:** Mathieu Roy, Ben Seymour, Susanne Becker, Clay Holroyd

**Affiliations:** a McGill University, Psychology, Montreal, Quebec, Canada; b Wellcome Centre for Integrative Neuroimaging, John Radcliffe Hospital, Headington, Oxford, UK; c University of Zurich, Department of Chiropractic Medicine, Zurich, Switzerland; d Department of Experimental Psychology, Ghent University, Ghent, Belgium

## Abstract

Pain fulfils a vital function, motivating behavior to avoid harm and ensure well-being. Pain therefore acts as a learning signal allowing organisms to predict future occurrences of pain and thus guiding escape and avoidance behavior. In line with research on reward processing and learning, recent research has highlighted central functions of the brain regions such as the anterior cingulate cortex (ACC), fronto-striatal brain circuits, and the neurotransmitter dopamine in mediating such motivational components of pain. Interestingly, in chronic pain – when pain loses its vital function as a warning signal – a shift away from nociceptive brain circuits and towards enhanced emotional-motivational processing has been described. This symposium aims to review current research on the brain mechanisms of the motivational component of pain and related learning. We consider computational models of the pain system and how decision-making aspects of pain might be involved in tuning the magnitude of the pain signal itself (i.e. endogenous pain control). Decision-making is based on motivation, which can be conflicting in case of co-occurring pain and reward leading to fine-tuned pain modulation. In this context, dopamine and fronto-striatal brain circuits play essential roles. These processes can also be seen in a broader context of error modelling. Correspondingly, we will present a computational model of the roles of ACC and dopamine in regulating effortful behavior in rodents, and an extension of this model to the domain of pain processing in humans.

**Speaker 1**: Ben Seymour, Ph.D., Wellcome Centre for Integrative Neuroimaging, John Radcliffe Hospital, Headington, Oxford, United Kingdom, ben.seymour@ndcn.ox.ac.uk

**Speaker 1Abstract Title**: Motivation, decision-making and endogenous control

Pain is often considered intangibly subjective, and theory has so far failed to capture the core essence of pain as a sensory and emotional percept. Since noxious stimulation usually leads to the perception of pain, pain has traditionally been thought of as sensory nociception, but its variability and sensitivity to a broad array of cognitive and motivational factors have led to a common view that it is inherently imprecise and intangibly subjective. However the core function of pain is motivational - to direct both short and long-term behaviour away from harm. Here I will show how an engineering approach allows us to reframe pain in terms of signal processing and control theory, and present a reinforcement learning model of pain. This offers a mechanistic understanding of how the brain supports pain perception and behaviour, illustrating the underlying computational architecture of the pain system in the brain. Importantly, it provides an explanation as to why pain is tuned by multiple factors and necessarily supported by a distributed network of brain regions, and so recasts pain as a precise and objectifiable control signal.

**Speaker 2**: Susanne Becker, PhD, University of Zurich, Department of Chiropractic Medicine, Zurich, Switzerland, Susanne.becker@balgrist.ch

**Speaker 2 Abstract Title**: Differential effects of reward on pain: perception, behavior and neural correlates

**Speaker 2 Abstract**: Pain and reward can be considered as the two ends of a hedonic continuum, with emotional-motivational functions as the common currency. Correspondingly, intersections between pain and reward have been described repeatedly, leading, for example, to reward-induced pain inhibition and cost-benefit analysis during decision making. However, reward does not cause a blanket inhibition of pain. Instead, pain perception can also be facilitated by certain types of decisions and pain discrimination appears spared by co-occurring reward. Here, I will discuss the manyfold interactions of pain and reward together with their underlying mechanisms. In this context, the neurotransmitter dopamine appears to play a central role. In contrast to common assumptions, dopamine’s role in pain is more complex than simple antinociceptive effects. Instead, dopamine appears to modulate pain motivation, facilitating either pain tolerance or avoidance depending on internal and external factors of an individual. Moreover, dopamine appears to act not only as a neurotransmitter but also a neuromodulator and as such mo dulating brain circuits associated with pain motivation such as fronto-striatal brain circuits. Altered functional connectivity in such fronto-striatal networks have been proposed to be a driver in the development of chronic pain and to represent a shift from nociceptive predominate emotional-motivational brain networks.

**Speaker 3**: Clay Holroyd, Ph.D. Department of Experimental Psychology, Ghent University, Ghent, Belgium, clay.holfoyd@ugent.be

**Speaker 3 Abstract Title**: No Pain, No Gain: Neurocognitive Mechanisms Supporting Effortful and Painful Behaviors

**Speaker 3 Abstract**: Many people will pause on the edge of a frigid body of water before plunging in. If they have committed to the action, then why do they hesitate? I propose that behaviors that result in discomfort require the boost of a cognitive control mechanism that attenuates these immediate costs (pain and effort), but not costs like monetary losses, to achieve longer-term goals (e.g., fitness). Fluctuations in control levels give rise to the conflicts observed when implementing these decisions. First, I will present a computational model based on principles of hierarchical reinforcement learning that simulates rat behavior in tasks involving physical effort and task switching. Key to the model are control signals at multiple levels of hierarchy that overcome decision-related costs associated with each immediately subordinate level. Attenuating the control signal at an intermediate level yields behavior that mirrors that of rats with anterior cingulate cortex lesions, which avoid effortful actions, and attenuating the control signal at a higher level mirrors the behavior of rats with prelimbic legions, which perseverate at task strategies. Then, I will present behavioral evidence in humans showing that choice behavior and response speeds are different when choosing actions with painful outcomes (electric shocks) compared to choosing actions that incur monetary losses, even when these types of aversive outcomes are equated for their subjective value using a calibration procedure. Further, a model that incorporates the control mechanism from the rat simulations accounts better for this choice behavior than do models based on standard principles of reinforcement learning.

**Learning Objective 1**: Re-conceptualize pain as a motivational signal rather than a sensation.

**Learning Objective 2**: Understand the interactions between pain and reward together with their underlying mechanisms.

**Learning Objective 3**: Examine the role of cognitive control when accepting pain in order to obtain rewards.

# Abstract: From CNS mechanisms of pain to pain biomarkers

**Authors:** Mathieu Roy, Christian Büchel, Tor D. Wager, Choong-Wan Woo

**Affiliations:** a McGill University, Department of Psychology, Montreal, Quebec, Canada; b Institut für Systemische Neurowissenschaften, Universitätsklinikum Hamburg-Eppendorf, Hamburg, Germany; c Dartmouth College, Hanover, NH, USA; d Sungkyunkwan University, Center for Neuroscience Imaging Research at IBS, South Korea

## Abstract

**Symposium Abstract**: Pain normally originates from nociceptive signals coming from the periphery and gradually making its way through the spinal cord, the brainstem, thalamus, cerebral cortex and subcortical structures. At all of these levels, this nociceptive signal is subjected to various sources of modulation and recent advances in neuroimaging now allows to examine pain processing at these various levels of the central nervous system. In the first part of this symposium, Dr. Büchel will discuss how the endogenous opioid system can modulate this system through expectations and past experience. Moreover, the teaching function of this nociceptive signal will be discussed in light of a recent study using a combination of high temporal (EEG) and high spatial resolution (fMRI) neuroimaging. Then, in the second part of this symposium, Dr. Wager will discuss what happens when nociceptive signals reach the brain and become interpreted as pain. Different types of pain lead to different characteristic pain-predictive patterns of brain activity, which share some similarities, but also important differences, with other types of non-painful affective experiences. Results from this line of research suggest that new brain-based ontologies are needed to measure and understand human pain and affect. Finally, in the last part of this symposium, Dr. Woo will discuss how this general approach can be applied to tonic and chronic pain. More specifically, results suggest that connectivity in the fronto-parietal attentional network is substantially affected by tonic pain. Altogether, this symposium will provide an overview of the CNS mechanisms that encode painful stimuli and predict subjectively reported pain in humans.

**Speaker 1**: Christian Büchel, Ph.D., Institut für Systemische Neurowissenschaften Haus W34, Universitätsklinikum Hamburg-Eppendorf Martinistr. 52, D-20246 Hamburg, Germany, buechel@uke.de.

**Speaker 1 Abstract Title**: What spinal and cortical signals can tell us about pain as an unpleasant sensation and teaching signal?

**Speaker 1 Abstract**: Pain is currently understood as the integration of peripheral nociceptive input with an internal state of the organism governed by current (e.g. expectation) and past (e.g. experience) information. This process is mainly implemented in the central nervous system comprising the spinal cord, brainstem and cerebral cortex. Recent advances in functional magnetic resonance imaging has allowed to investigate this system as a whole and enabling system level investigations in humans. This presentation will start with the basic mechanisms of pain processing of phasic experimental heat stimuli in the spinal cord, brainstem and cortex. In a second step studies will be presented showing how the endogenous opioid system can modulate this system through expectation and experience. In addition, these results will be compared to a pharmacological challenge with remifentanil. Importantly, this presentation will also address the question of whether certain aspects of these mechanisms pertain to pain per se or are just unspecific consequences of the high salience of nociceptive input. Apart from its perceptual characteristics, pain is also a strong teaching signal to guide future behavior. Using a combination of high temporal (EEG) and high spatial resolution (fMRI) neuroimaging, we will show the spatial and temporal orchestration of this teaching signal. Finally, the presentation will focus on novel approaches to extend these insights to tonic pain stimuli and thus bringing them closer to the clinical reality of chronic pain.

**Speaker 2 Abstract Title**: What has neuroimaging taught us about the neural architecture for evoked pain and emotion?

**Speaker 2 Abstract**: We often assume that brain organization conforms to our psychological categories. For example, brain representations for pain, social rejection, guilt, and fear ought to be similar, as all of them are “negative” and motivate escape and avoidance. Early neuroimaging studies seemed to confirm these theories, identifying common brain activations across pain and other types of negative experiences. Recent studies, however, have overturned this view, and suggest that these early findings arose from a combination of selective interpretation of evidence and focus on non-selective brain signals that respond to many types of tasks (not just aversive or “painful” ones). These studies have several advantages: (1) multivariate pattern analysis to identify patterns that predict experience, rather than simply responding to task demands; (2) unbiased analyses of similarities and differences between somatic pain and other affective challenges; (3) comparison of effect sizes, rather than yes/no hypothesis tests; (4) construct validation approaches designed to identify common and distinct brain patterns across multiple types of pain and affect; and (5) larger sample sizes than early studies, with internal validation on independent cohorts. Collectively, these studies reveal three broad conclusions about the neural architecture for pain and affect. First, brain representations of evoked pain are largely distinct from representations of other affective processes. Second, pain-processing systems share a common core that generalizes across multiple types of painful stimuli (evoked visceral, cutaneous thermal, and cutaneous mechanical). And third, different types of affective challenges evoke different (but reproducible across individuals) patterns of brain activity—the similarities among which do not reflect current psychologically based ontologies. At the brain level, different types of negative emotional experiences are not interchangeable, suggesting that new brain-based ontologies are needed to measure and understand human pain and affect.

**Speaker 3 Abstract Title**: Dynamic Reconfiguration of Functional Brain Networks During Sustained Pain

**Speaker 3 Abstract**: Pain is not just a simple reflex to nociceptive input, but it is rather constructed through complex interactions among multiple brain systems over time. However, it remains poorly understood how the patterns of functional brain connectivity dynamically change over the period of experiencing sustained pain. In this talk, I will introduce two functional Magnetic Resonance Imaging (fMRI) studies in which we tried to understand the dynamic changes in functional brain connectivity and its community structures while participants were experiencing tonic pain. The results suggest that the top-down cognitive and affective regulatory brain systems are important for explaining tonic pain experience, and particularly the fronto-parietal network serves as a flexible hub modulating both the early attention to pain and also late regulation of pain, playing key roles in orchestrating multiple brain networks during sustained pain. Overall, our findings provide a new insight into how dynamic interactions among multiple brain systems construct and process pain experience over time, which could potentially advance our mechanistic understanding of sustained and clinical pain.

**Learning Objective 1**: To understand how the endogenous opioid system can modulate the transmission of nociceptive signals through expectation and experience.

**Learning Objective 2**: To examine how the patterns of brain activity that predict pain and other emotions can provide information on the degree of similarity between these different affective experiences.

**Learning Objective 3**: To understand how functional connectivity can predict tonic and chronic pain.

# Abstract: The social neuroscience of empathy for pain and touch

**Authors:** Inge Timmers, Marina López-Solà, Loren J. Martin

**Affiliations:** a Stanford University, Department of Anesthesiology, Perioperative, and Pain Medicine, Palo Alto, CA, USA; b University of Barcelona, Unit of Psychological Medicine, School of Medicine, Barcelona, Spain; c University of Toronto Mississauga, Department of Psychology, Mississauga, ON, Canada

## Abstract

Pain is considered a personal experience, but it is, in fact, rarely private. Individuals’ behavioural responses to pain function to communicate distress to others in the environment, eliciting emotional reactions and caregiving actions that will, in turn, impact the sufferer’s pain experience. This symposium will highlight the importance of understanding the social environment in modulating pain responses so that novel pain management strategies may be developed. Together, we will present state-of-the-art tools and techniques that will help to bridge the gap between human and animal knowledge to tackle the questions of causality between different neuronal populations, perception, and behavior in rats, mice, and humans. Evidence from both the basic science and clinical perspectives will be presented, illustrating how painful experiences can impact social interactions and how reactions from others in the social environment and the environment itself impact the sufferer’s pain experience. Given the complex nature of social context and social interactions on pain sensitivity in animals and people, dissecting their integral role in mediating pain outcomes is critical.

We propose a symposium to discuss the important influence of social interactions and context on pain responses and empathic behaviour in mice and people. In addition, we will discuss issues related to the feasibility and conduct of human as well as animal studies examining questions pertinent to the social modulation of pain. Our goal is to engage clinicians with pain neuroscientists to address how the community can best address these complex questions.

**Speaker 1**: Inge Timmers, PhD, Stanford University, Department of Anesthesiology, Perioperative, and Pain Medicine, Palo Alto, CA, USA, itimmers@stanford.edu, @Inge__Timmers

**Speaker 1 Abstract Title**: Parental responses to their child’s pain: the role of empathy

**Speaker 1 Abstract**:

We have all experienced the distress of observing someone else in pain, but it is particularly difficult for a parent to see their child suffering. Research shows that, in the context of pediatric chronic pain, parent pain catastrophizing, fears, maladaptive responses to their child’s pain, and modeling of pain behaviors may negatively impact children’s pain coping and pain-related functioning. A key variable that thus far has been largely neglected is parent affective responses, and more specifically parent empathic distress. This empathic distress response is fast, automatic, and can be overwhelming, and may result in the hindering of the parent’s ability to respond in ways to promote the child’s adaptive functioning. Dr. Timmers will present her research on parent empathic distress in the context of pediatric chronic pain. She will present on relations between parent empathic distress and other relevant parent variables (e.g., protective responses, pain catastrophizing) as well as child functioning. Also, she will present data from an experimental study examining how a parent’s brain and body responds to observing their own child in pain, using an empathy paradigm that harnesses each child’s personal and unique pain experiences.

**Speaker 2**: Marina López-Solà, PhD, Serra Hunter Programme, University of Barcelona, Unit of Psychological Medicine, School of Medicine, Barcelona, Spain, mlopezsola@ub.edu, @mlopezsola82

**Speaker 2 Abstract Title**: Brain mechanisms of social touch-induced analgesia and prosocial transformation of the meaning of pain

Speaker 2 Abstract:

Supportive touch and transformations in the meaning we attribute to pain have remarkable benefits during painful medical procedures and in the context of childbirth. But do social touch and prosocial transformations in the meaning of pain influence pain neurophysiology, i.e., the brain processes linked to nociception and primary pain experience? What other brain processes beyond primary pain systems mediate these analgesic effects? In this session, Dr. López-Solà will cover how social touch and socially-induced transformations of the meaning of pain affect pain perception across a variety of studies. She will offer an overview on the psychological and neurophysiological mediators of such effects. And, she will explain the effects of handholding and transformations of the meaning of pain on the Neurologic Pain Signature (NPS) -a multivariate brain pattern sensitive and specific to somatic pain- and on other brain systems that are relevant for conferring sensory, affective and value qualities to the pain experience. She will provide novel insights on how social touch and meaning transformation affect core brain processes that contribute to pain and pain-related distress, and how these strategies should be considered alongside other treatments in medical and caregiving contexts.

**Speaker 3**: Loren J. Martin, PhD. Assistant Professor, University of Toronto Mississauga, Department of Psychology, Mississauga, ON Canada, lj.martin@utoronto.ca, @_ljmartin

**Speaker 3 Abstract Title**: Mechanistic insight into the social modulation of pain behavior

**Speaker 3 Abstract**: Pain is often thought of as a sensory experience, but it has a distinct emotional aspect to it. This is no more evident than the thoughts and feelings that we experience when we watch another person in pain. The observation of another person in pain elicits similar emotional responses as the firsthand experience of pain and as such, pain is often used to measure empathy. The naturalistic basis for empathy is built upon simplistic behaviors such as emotional contagion and state-matching and we have shown that pain contagion is inhibited through a coordinated stress response in unfamiliar (i.e. stranger) pairs – both mice and people. Dr. Martin will discuss his research on modulation of pain contagion by social familiarity and the role of the stress response using both mouse and human models. His lab has recently completed a brain-wide histological investigation of pain contagion (in mice), coupled with pharmacological, behavioral and slice physiology studies, that point toward a novel cortical mechanism for the suppression of pain contagion. In addition, he will discuss the activation of specific neural circuits that are activated through social interactions and work to suppress pain and anxiety.

**Learning Objective 1**: Upon completion of this session, attendees will be able to discuss the different components of empathy in the context of pain across different species

**Learning Objective 2**: Upon completion of this session, attendees will be able to discuss the physiological underpinnings of physical social contact and its contribution to the modulation of pain

**Learning Objective 3**: Upon completion of this session, attendees will be able to reflect on the role of empathy in parent-child interactions in the context of pain

# Abstract: Using digital technologies for pain management and education across the age spectrum: experiences from three provinces

**Authors:** Susan Tupper, Jennifer Stinson, Samina Ali

**Affiliations:** a Saskatchewan Health Authority, Department of Safety, Quality & Clinical Standards, Saskatoon, Saskatchewan, Canada; b The Hospital for Sick Children and the Lawrence S Bloomberg Faculty of Nursing, University of Toronto, Toronto, Ontario, Canada; c University of Alberta, Departments of Pediatrics & Emergency Medicine, Edmonton, Alberta, Canada

## Abstract

Digital health technologies are innovative approaches that can promote better health and learning outcomes in patients, family caregivers, and healthcare providers. This symposium will explore the development of digital health technologies to improve pain management and education in hospitalized children, children in the emergency department, youth with chronic pain, and adults with dementia. Presenters will speak to the importance of working collaboratively with developers of digital health technologies (programmers) and using a user-centred design approach that includes needs assessments, co-designing with patient partners, usability and feasibility testing, and implementation phases. This workshop will include virtual reality, humanoid robotics, tablet technology, and smartphone applications. The opportunities and challenges of using digital health technologies in every day clinical practice will be discussed.

**Speaker 1**: Susan Tupper, PT, PhD, Saskatchewan Health Authority, Quality Safety & Clinical Standards, Saskatoon, Saskatchewan, Canada, susan.tupper@usask.ca, @smtpt

**Speaker 1 Abstract Title**: Virtual reality for pain management education of family caregivers and people living with dementia

**Speaker 1 Abstract**: Although pain is common in adults with dementia, many misconceptions exist among family caregivers and healthcare providers due to the impact of dementia on pain expression. Family caregivers play multiple roles in supporting pain management in the home and healthcare settings, such as advocating for diagnosis and care, knowledge brokers between family and care providers, and supporting day to day pain management activities. Due to their unique knowledge of the person living with dementia, family caregivers are key healthcare decision makers about pain management, yet there are few opportunities for family caregivers to learn how to support management of pain in people affected by dementia. Virtual reality training is a novel approach to education delivery for family caregivers and people living with dementia. In addition, experiential virtual reality videos offer unique treatment opportunities. Dr. Tupper will describe findings from: 1) interviews with people living with dementia and their family caregivers to identify pain assessment and management learning needs, 2) prioritization of learning topics to co-design a pain education learning curriculum and 360° virtual reality videos, 3) end-user perspectives on educational and experiential virtual reality videos.

**Speaker 2**: Jennifer Stinson, RN-EC, PhD, CPNP, FAAN, The Hospital for Sick Children and the Lawrence S Bloomberg Faculty of Nursing, University of Toronto, Toronto, Ontario, Canada, Jennifer.stinson@sickkids.ca, @DrJenStinson

**Speaker 2 Abstract Title**: Into the future: How digital health technologies can reduce pain and distress in hospitalized children

**Speaker 2 Abstract**: Acute pain is commonly experienced by hospitalized children and can have short and long term negative consequences. New technologies, such as smartphone apps, virtual reality (VR) and robotics, have allowed for the development of novel and effective distraction techniques for children and adolescents. Dr. Stinson will discuss a user centred design approach to develop and evaluate (a) a smartphone app called Achy Penguin for post-operative pain co-designed and tested with a parent partner, and (b) a humanoid robot and an underwater VR program for children and youth with cancer undergoing subcutaneous port access. The studies included: (1) the testing of the usability and safety of the technology and (2) preliminary effectiveness of the technology through pilot randomized controlled trials (RCT). She will also discuss the opportunities and challenges of implementing these technologies in everyday clinical practice.

**Speaker 3**: Samina Ali, MDCM, FRCPC, University of Alberta, Departments of Pediatrics & Emergency Medicine, Edmonton, Alberta, Canada. sali@ualberta.ca, @drsaminaali

**Speaker 3 Abstract Title**: Harnessing digital technology to reduce children’s distress and pain in the emergency department and beyond

**Speaker 3 Abstract**: Close to 2.5 million children visit Canadian emergency departments (EDs) each year for the treatment of potentially painful injuries and illnesses. When hospitalized, almost 80% of children will undergo several (average 6.3) procedures per day and many will experience moderate to severe pain. Poorly managed pain adversely impacts a child’s wellbeing and contributes to a reduced ability to cope effectively with future pain. Digital technology distractors are pervasive in society, but their uptake in pain care is still limited. Almost all major Canadian pediatric EDs have access to some type of digital tool, but ED physicians do not report regular use of these to treat or prevent pain.

Dr. Ali will present the results of recently conducted systematic reviews of digital technology in healthcare and present her recently conducted trials of digital technology (iPads, humanoid robots, virtual reality) for procedural pain in the ED. She will also discuss an ongoing international, transdisciplinary collaboration to develop a more socially responsive robot for pain care. Given her clinical and research experience with this technology, she is uniquely positioned to comment on the feasibility, utility, and unique challenges of these techniques in the acute care environment. Join her to decide what works better in your setting … robots or bubbles!

Learning Objective 1:

Describe development and user perspectives of a novel virtual reality 360° video application for pain education of family caregivers and adults with dementia.

Learning Objective 2:

Describe a user-centred design approach to develop and evaluate virtual reality, humanoid robots and smartphone apps for pain management in pediatrics.

Learning Objective 3:

Name 3 effective digital techniques to reduce children’s pain and distress of minor medical procedures.

# Abstract: Quantitative Sensory Testing in Pain Research: Methods, Applications, and Future Directions in Populations Across the Lifespan

**Authors:** Perri R. Tutelman, Javeria Ali Hashmi, Roger B. Fillingim

**Affiliations:** a Dalhousie University, Psychology and Neuroscience, Halifax, NS, Canada; b Dalhousie University, Department of Anesthesia, Pain Management & Perioperative Medicine, Halifax, NS, Canada; c University of Florida, Pain Research & Intervention Center of Excellence, Gainesville, FL, USA

## Abstract

**Symposium Abstract**: Quantitative Sensory Testing (QST) is a non-invasive technique that has been used extensively in pain research over the past 3 decades. The term QST refers to a set of procedures that assess perceptual responses to the application of standardized sensory stimuli with the goal of assessing somatosensory function. QST has significantly advanced our understanding of the neurobiological mechanisms and psychosocial influences that underpin typical and atypical sensory processing, which in turn, has aided in our understanding of pain conditions and the identification and refinement of tailored pain therapies. However, the application of QST in pain research has seen challenges such as variability in the methods used and failure to harness the full potential of what QST can offer across paradigms and populations. This workshop will provide a comprehensive overview of QST methods and applications and will discuss priority areas for future research. In this workshop, an international panel of speakers will present on the utility and practical use of traditional (e.g., sensory profiling) and advanced (e.g., pain modulation, functional neuroimaging) QST paradigms for inductive and deductive pain inquiry. Data demonstrating the application of various QST techniques to populations across the lifespan (e.g., pediatrics, adults, and older adults), including key knowledge gaps in these respective areas, will be discussed. Procedural modifications to allow for safe QST testing amidst the global COVID-19 pandemic, including with high risk populations (e.g., older adults), will be discussed. Finally, this workshop will offer perspectives on opportunities for meaningful patient and family engagement in QST studies.

**Speaker 1**: Javeria Ali Hashmi, PhD, Dalhousie University, Department of Anesthesia, Pain Management & Perioperative Medicine, Halifax, NS, Canada, Javeria.Hashmi@dal.ca, @netphys1

**Speaker 1 Abstract Title**: Individual Differences in Pain Sensitivity; Interplay Between Nociception and Top-down Mental Factors

**Speaker 1 Abstract**: Pain perception is distinct from nociception or simple sensing. It is a combination of prior mental states that represent top-down perceptual processes and bottom-up nociceptive features. Top-down components can be implicit such as conditioned aversive effects or are explicit mental constructs such as expectations, beliefs and conceptual schemas. Pain perception is thus a combination of sensing, organizing and interpreting of noxious inputs based on context and prior knowledge. Top-down modulation of perceived pain has been reliably documented by changing expectations. On the other hand, simple quantitative sensory testing for observing pain behavior has limitations since it theoretically quantifies the sensing aspects of pain response but is not amenable to directly measuring responses that are mediated by top-down factors. From this vantage point, psychosocial aspects such as gender identity and self-schemas are not fully captured in QST tests on pain sensitivity. In this talk, I will discuss QST approaches that allow deductive inquiries to assess bottom-up and top-down pain processes along with study of mediating brain mechanisms when used in combination with neuroimaging. Sex and gender research on pain will be discussed as an example to illustrate potential caveats and pitfalls of ignoring effects of top-down brain processes on pain. This talk will offer suggestions on how existing controversies related to inconsistent findings of high pain sensitivity in females can be resolved through deductive inquiries into effects of top-down factors on pain perception.

**Speaker 2**: Roger B. Fillingim, PhD, University of Florida, Pain Research & Intervention Center of Excellence, Gainesville, FL, USA, rfilling@ufl.edu

**Speaker 2 Abstract Title**: Using QST to Investigate Pain Modulation in Older Adults

**Speaker 2 Abstrac**: Older adults are at greater risk for chronic pain and pain-related disability compared to their younger counterparts. While these age-related increases in pain risk are often attributed to cumulative wear and tear, considerable evidence demonstrates that aging is associated with altered pain processing, which may also contribute to the greater burden of clinical pain later in life. While traditional quantitative sensory testing (QST) measures (e.g. pain threshold) demonstrate minimal age-related changes, this presentation will review findings from recent studies using more sophisticated QST approaches that reveal maladaptive changes in pain modulatory balance. Specifically, older adults exhibit increased pain facilitation and reduced pain inhibition compared to younger adults. In addition, pain conditions that are more common in older adults have been associated with altered pain processing. The presenter will discuss recent research in older adults with knee osteoarthritis showing a generalized enhancement of pain sensitivity compared to age-matched controls without knee OA. The presentation will conclude with a summary of these QST findings in older adults and a discussion of how QST can be incorporated into future research to elucidate age-related changes in pain processing.

**Speaker 3**: Perri R. Tutelman, BHSc (Hons.), Dalhousie University, Psychology and Neuroscience, Halifax, NS, Canada, ptutelman@dal.ca, @PerriTutelman

**Speaker 3 Abstract Title**: Experimental Applications of QST in Pediatric Pain Research

**Speaker 3 Abstract**: While QST has been used extensively in adult pain research for several decades, it has been more recently applied to pediatric populations. QST offers numerous advantages over other experimental pain paradigms commonly used in pediatric pain research (e.g., the cold pressor task), however uncertainty remains regarding the use of QST with children including the acceptability and feasibility of the tasks, ethical considerations associated with the procedures, and the complexity of somatosensory development across childhood. This presentation will provide an overview of the use of QST in pediatric populations and will highlight priority areas for future research. First, results from a new scoping review mapping the current state of QST research in pediatric pain will be presented, including the QST paradigms and procedures used with children to date, and the characteristics of pediatric populations to which QST has been applied. Key empirical and methodological gaps identified in the literature on the use of QST in children will be discussed. Next, common ethical and methodological issues associated with the use of QST in children will be reviewed. Strategies for mitigating these challenges will be discussed using acceptability and feasibility data from recent sensory profiling studies with healthy (ages 8-15 years) and clinical (childhood cancer survivors; ages 8-17 years) pediatric populations. Videos demonstrating QST procedures adapted for children will be presented. Finally, this presentation will highlight opportunities for the meaningful engagement of patients and families across the stages of QST studies, and our experience with the logistics and benefits of doing so.

**Learning Objective 1**: To describe the utility and practical use of traditional and advanced QST paradigms in pain research.

**Learning Objective 2**: To understand how QST can be applied to pain research with populations across the lifespan.

**Learning Objective 3**: To consider current controversies and future directions regarding the use of QST in pain research.

# Abstract: The role of altered cognitive processing of bodily sensations in pain: A need for innovative and integrative research

**Authors:** Dimitri M.L. Van Ryckeghem, Aline Wauters, Tanja Hechler

**Affiliations:** a Section Experimental Health Psychology, Clinical Psychological Science, Faculty of Psychology and Neuroscience, Maastricht University, Maastricht, The Netherlands; b Department of Experimental-Clinical and Health Psychology, Faculty of psychology and Educational Sciences, Ghent University, Ghent, Belgium; c Department of Clinical Psychology and Psychotherapy for Children and Adolescents, University of Trier, Trier, Germany

## Abstract

**Symposium Chair:** Dimitri M.L. Van Ryckeghem Assistant Professor, Section Experimental Health Psychology, Clinical Psychological Science, Faculty of Psychology and Neuroscience, Maastricht University,Maastricht, The Netherlands. Email: Dimitri.VanRyckeghem@maastrichtuniversity.nl Twitter: @dvryckeg

**Symposium Abstract**: For decades, researchers have been investigating how altered cognitive processing of internal and external bodily sensations impact upon pain experience as well as related levels of distress and disability. In particular, biases in attention, interpretation and memory of both internal and external bodily sensations have been thought to influence the outcome and maintenance of pain.

Yet, despite a long track record and the many advances made in this domain in understanding the impact of cognitive processing of bodily sensations upon pain outcomes, several questions remain unanswered. Within current symposium, we aim to highlight existing gaps in available knowledge in this field and call for innovative research systematically investigating the link between altered processing of attention, interpretation and memory for both internal and external bodily sensations rather than research investigating isolated phenomena.

In particular, we will discuss the role of altered interoception and cognitive processing of pain in experiencing and maintaining pain in adults and children. Speakers will argue for the need of integration when discussing the impact of attention, interpretation and memory biases upon pain outcomes as well as the need of integration of cognitive processing of internal and external bodily sensations in the context of pain using ecological valid and innovative research designs.

**Speaker 1**: Dimitri M.L. Van Ryckeghem Assistant Professor, Section Experimental Health Psychology, Clinical Psychological Science, Faculty of Psychology and Neuroscience, Maastricht University, Maastricht, The Netherlands. Email: Dimitri.VanRyckeghem@maastrichtuniversity.nl Twitter: @dvryckeg

**Speaker 1 Abstract Title**: Cognitive biases for pain: A functional-contextual perspective.

**Speaker 1 Abstract**: Cognitive biases – i.e., selectively attending to pain-related information (attention bias), interpreting ambiguous pain and/or health relevant information as threatening (interpretation bias), and/or recalling pain-related information as more negative/threatening than initially experienced (memory bias), have been theorized to be key in explaining poor pain outcomes. Typically, existing models assume that cognitive biases are maladaptive, trait-like processes. However, research findings are inconsistent and puzzling, with mixed support for theoretical-driven assumptions. Within this lecture, Dimitri Van Ryckeghem will review the cognitive bias literature and discuss the need for a radical conceptual shift towards an integrated functional-contextual perspective in current understanding of cognitive biases, focusing on the adaptive character of cognitive biases and the need for research to consider goal pursuit and context variables. Using an innovative virtual-reality approach, increasing ecological validity, he will present research where he bridges the gap between the lab and daily life, allowing the investigation of cognitive biases and its dynamics in real-life contexts, elucidating how contextual and goal-relevant factors influence cognitive processing of pain and discuss implications for clinical practice.

**Speaker 2**: Aline Wauters (MsC) Department of Experimental-Clinical and Health Psychology, Faculty of psychology and Educational Sciences, Ghent University, Ghent, Belgium, Email: Aline.wauters@ugent.be

**Speaker 2 Abstract Title**: Linking child attentional bias and memory bias in the context of pediatric pain: how parental talk might be of influence

**Speaker 2 Abstract**: Both negatively biased pain memories (i.e., recalling pain as higher than initially reported) and attentional biases towards pain-related information are considered cognitive factors implicated in pain trajectories. While research suggests that both biases share linked mechanisms of action, insight into how pain-related attention and memory for pain influence each other is lacking. Research shows that how parents talk to their children influences child memory in the context of pediatric pain. Drawing upon existing research, parental talk might contribute to child pain memories through its influence on child attention to pain. Parents might talk in a pain attending or either non-pain attending way to their child about future and past pain experiences, but might also differ in the narrative style they use when talking to their child (i.e., elaborative vs repetitive style). Data investigating the role of (the various aspects of) parental talk in explaining the relationship between child attention for pain and child pain memories as well as child pain expectancies will be presented. These relationships will be explored in both a healthy school sample and a clinical sample with children suffering chronic pain.

**Speaker 3**: Tanja Hechler (PhD) Address: Department of Clinical Psychology and Psychotherapy for Children and Adolescents, University of Trier, Trier, Germany Email: hechler@uni-trier.de Twitter: @HechlerTanja

**Speaker 3 Abstract Title**: Altered interoception in children and adolescents with chronic primary pain: An overview and call for action.

**Speaker 3 Abstract**: Interoception is defined as the process by which the nervous system senses, interprets, and integrates internal bodily sensations, providing a moment-to-moment mapping of the body’s internal landscape. Today, interoceptive sensations are regarded as conscious percepts that result from a constructive process, in which the brain interprets information from the body in light of predictions given past experience. Clearly, top-down influences such as cognitive processes - learning, memory and cognitive biases - need to be considered when understanding conscious interoception.

In adults, altered interoception, i.e. altered ways in how and how well adults perceive their internal body state, is today considered a prominent feature of a variety of both, mental health and chronic primary pain, and even discussed as a common *p*-factor that subsumes features of various mental health conditions. Research is clearly lacking behind in children with chronic primary pain.

Within this presentation, we argue for the integration of (altered) interoception into theoretical models accounting for the co-occurrence of mental health problems and chronic primary pain in children. First, we describe some characteristics of interoception, relevant for pain research in children. Second, we summarize evidence for altered interoception from experimental studies, such as altered interoceptive accuracy, altered emotional responses and cognitive processing, where existent. Third, we outline a developmental pathway on how altered interoception might develop, including etiological factors that we consider relevant for prevention and treatment such as sensitive periods for altered interoception, autonomic dysfunction, and altered parental interoception and emotion regulation.

**Learning Objective 1**: Delegates will gain understanding in the dynamic and functional nature of cognitive biases, driven by goals and contextual information.

**Learning Objective 2**: Delegates will gain insight in the interplay between attention, interpretation and memory biases and their combined impact upon pain outcomes in children and adults.

**Learning Objective 3**: Delegates will gain insight concerning the impact of altered interoception upon pain experience, disability and distress in children and adults with (chronic) pain.

# Abstract: Disparities in child pain care: Antecedents, consequences and underlying mechanisms.

**Authors:** Tine Vervoort, Megan Miller, Lindsey Cohen, Fleur Baert

**Affiliations:** a Ghent University, Department of Experimental-Clinical and Health Psychology, Ghent, Oost-Vlaanderen, Belgium; b Indiana University – Purdue University Indianapolis, Department of Psychology, Indianapolis, IN, USA; c Georgia State University, Department of Psychology, Atlanta, GA, USA

## Abstract

**Symposium Chair**: Tine Vervoort, PhD, Ghent University, Department of Experimental- Clinical and Health Psychology, Ghent, Oost-Vlaanderen, Belgium, tine.vervoort@ugent.be, @tinevervoort13.

**Symposium Abstract**: A growing body of research in pediatric pain suggests that under certain circumstances observers (e.g., parents, clinicians, …) may engage in discriminatory behaviors when facing or caring for a child with pain. This may lead to disparities in pediatric pain care which can, in turn, substantially impact children’s pain experience, recovery and general functioning. Several research groups have focused on understanding the antecedents, consequences and underlying mechanisms of such disparities in child pain treatment, which will be the topic of this symposium. First, Dr. Megan Miller will discuss the influence of patient race on provider pain- related attention, which may help explain racial disparities in pediatric pain management. She will present a recently completed study using eye-tracking technology to investigate how providers’ visual attention varies across pediatric patient race during a pain assessment task. Dr. Lindsey Cohen will then discuss how a child’s perceived gender may bias adult ratings of the child’s pain severity, potentially leading to biased clinical decision making and thus contributing to gender disparities in child pain care. Lastly, Fleur Baert will consider how such disparities may affect both the child and their significant others (e.g., parents). Disparities in pain care are likely to elicit parental appraisals of injustice in response to the child’s pain and associated medical or interpersonal treatment. Fleur Baert will discuss how these appraisal processes may impact on the child’s pain experience through parental attentional and emotional processing and parental caregiving behavior.

**Speaker 1**: Megan Miller, PhD, Indiana University – Purdue University Indianapolis, Department of Psychology, Indianapolis, Indiana, US, meg.marie.miller@gmail.com, @megmarie_miller.

**Speaker 1 Abstract Title**: The influence of patient race on provider pain-related attention.

**Speaker 1 Abstract**: Pain demands attention. When assessing a patient with pain, healthcare providers visually attend to the patient to acquire information that is integral to determining treatment. In the broader non-pain literature, visual attention of the observer has been found to fluctuate based on a variety of factors, including the race of the person being attended to. Previous research has identified racial disparities in pediatric pain care, such that Black children are less likely to receive an analgesic than their White counterparts. These disparities may be driven, in part, by systematic differences in providers’ visual attention when assessing patients. Dr. Miller will review existing literature on pain-related attention, race differences in attention, and race disparities in pain care. In this context, Dr. Miller will present results of a recently completed study that used eye-tracking technology to investigate how providers’ visual attention varies across pediatric patient race during a pain assessment task. Fixation and gaze differences (ie, order, location, and length of gaze) across patient race, as well as the relationships between provider attention, implicit pain- related attitudes, and provider pain beliefs will be discussed. The presentation will conclude with future directions for this line of research.

**Speaker 2**: Lindsey Cohen, PhD, Georgia State University, Department of Psychology, Atlanta, Georgia, US, llcohen@gsu.edu, @CHAMPLab.

**Speaker 2 Abstract Title**: Adult rating of acute pediatric pain: The influence of child gender.

**Speaker 2 Abstract**: Adults are often tasked with judging children’s pain severity to determine significance and treatment course. These parents, nurses, or other adults make important clinical decisions based children’s pain behavior. However, judgement is comprised of accuracy and error, and error can be random or systematic. To remove or reduce systematic error, it must be identified and quantified. The purpose of these studies was to examine whether children’s gender systematically influences adults’ ratings of children’s pain. Although there are minimal if any differences between boys’ and girls’ pain sensitivity or behavior prior to adolescence, ample literature suggests that adults attribute characterological distinctions based on sex. We held children’s behavior constant while manipulating whether the adults who were completing pain ratings believed the child was a boy or a girl. In short, we deceived participants by stating that the child in the video was “Samuel” or “Samantha”; in fact, it was the same video of a 7-year- old child dressed in gender neutral clothing. Data indicate the deception was effective. In study 1, pain ratings for the undergraduates and nursing students (n = 84) of Samuel were higher than the ratings (n = 99) of Samantha. In study 2, 264 participants were randomized, and pain was higher for those (n = 133) rating Samuel than those (n = 131) ratings Samantha. These results are consistent with data showing that adults falsely believe young boys are more stoic and girls more emotional, sensitive, and expressive. This finding has a host of clinical implications.

**Speaker 3**: Fleur Baert, Msc, Ghent University, Department of Experimental-Clinical and Health Psychology, Ghent, Oost-Vlaanderen, Belgium, fleur.baert@ugent.be, @fleurasinfleur.

**Speaker 3 Abstract Title**: “It’s not fair!” The impact of parental injustice appraisals in response to child pain upon parental attention to anger and parental behavior.

**Speaker 3 Abstract**: Justice or equity has been conceptualized as a basic human motive and mounting evidence suggests that perceptions of (in)justice regarding one’s experiences of pain play a key role in physical and psychological adjustment in both adult and pediatric populations. Although injustice-relevant themes have long been present in clinical discourse, only recently has research addressed the role of injustice appraisals within health and pain outcomes. Further, recent advances in research have increasingly highlighted that observers’ appraisals of sufferers’ pain and associated behaviors significantly impact sufferer pain experience. These dynamics are particularly critical in highly dependent relationships, such as that between parent and child. Fleur Baert will discuss recent qualitative findings demonstrating the centrality of parental injustice appraisals in their experience of pediatric pain as well as in seeking pain care and how these entail appraisals on behalf of the child (child-oriented injustice appraisals) as well as the parent (parent-oriented injustice appraisals). Drawing upon self-report findings and new lab- based observational findings, Fleur Baert will further discuss how child- and parent-oriented injustice appraisals differentially impact child pain-related outcomes, parental attentional processing of personally relevant facial anger and pain cues and parental caregiving behaviour in response to child pain. Current evidence will be critically reviewed with discussion of future empirical and clinical directions.

**Learning Objective 1**: Attendees will come to understand how racial, sex- and gender-related factors contribute to profound disparities in pain care amongst pediatric populations.

**Learning Objective 2**: Attendees will come to understand how observer attentional processing and stereotype-based biases may influence their assessment of a child’s pain experience, which may provide key mechanisms underlying these disparities.

**Learning Objective 3**: Attendees will come to understand how such disparities in pediatric pain management may elicit appraisals of injustice both in the child as in their significant other(s) or caregivers (e.g., parents), which may further impact the child’s pain-related outcomes.
